# *De Novo* Assembly and Characterization of Four Anthozoan (Phylum Cnidaria) Transcriptomes

**DOI:** 10.1534/g3.115.020164

**Published:** 2015-09-17

**Authors:** Sheila A. Kitchen, Camerron M. Crowder, Angela Z. Poole, Virginia M. Weis, Eli Meyer

**Affiliations:** Department of Integrative Biology, Oregon State University, Corvallis, Oregon 97331

**Keywords:** coral, phylogenomics, nonmodel system, database

## Abstract

Many nonmodel species exemplify important biological questions but lack the sequence resources required to study the genes and genomic regions underlying traits of interest. Reef-building corals are famously sensitive to rising seawater temperatures, motivating ongoing research into their stress responses and long-term prospects in a changing climate. A comprehensive understanding of these processes will require extending beyond the sequenced coral genome (*Acropora digitifera*) to encompass diverse coral species and related anthozoans. Toward that end, we have assembled and annotated reference transcriptomes to develop catalogs of gene sequences for three scleractinian corals (*Fungia scutaria*, *Montastraea cavernosa*, *Seriatopora hystrix*) and a temperate anemone (*Anthopleura elegantissima*). High-throughput sequencing of cDNA libraries produced ∼20–30 million reads per sample, and *de novo* assembly of these reads produced ∼75,000–110,000 transcripts from each sample with size distributions (mean ∼1.4 kb, N_50_ ∼2 kb), comparable to the distribution of gene models from the coral genome (mean ∼1.7 kb, N_50_ ∼2.2 kb). Each assembly includes matches for more than half the gene models from *A. digitifera* (54–67%) and many reasonably complete transcripts (∼5300–6700) spanning nearly the entire gene (ortholog hit ratios ≥0.75). The catalogs of gene sequences developed in this study made it possible to identify hundreds to thousands of orthologs across diverse scleractinian species and related taxa. We used these sequences for phylogenetic inference, recovering known relationships and demonstrating superior performance over phylogenetic trees constructed using single mitochondrial loci. The resources developed in this study provide gene sequences and genetic markers for several anthozoan species. To enhance the utility of these resources for the research community, we developed searchable databases enabling researchers to rapidly recover sequences for genes of interest. Our analysis of *de novo* assembly quality highlights metrics that we expect will be useful for evaluating the relative quality of other *de novo* transcriptome assemblies. The identification of orthologous sequences and phylogenetic reconstruction demonstrates the feasibility of these methods for clarifying the substantial uncertainties in the existing scleractinian phylogeny.

Transcriptome sequencing provides a rapid and cost-effective approach for gene discovery in nonmodel organisms. Analysis of transcriptomes from a diverse range of invertebrates such as sponges (Riesgo *et al.* 2014; Conaco *et al.* 2012), ctenophores (Ryan *et al.* 2013), annelids (Riesgo *et al.* 2012), and mollusks (Riesgo *et al.* 2012; Kocot *et al.* 2011) has enhanced comparative and evolutionary studies of metazoans. Quantitative analysis of these sequences (RNA-Seq) has become the method of choice to profile genome-wide transcription levels. This technique provides an unbiased approach to discovering functional processes through identification and quantification of differentially expressed genes between phenotypic states including experimental treatments (Meyer *et al.* 2011), tissue types (Siebert *et al.* 2011), and developmental stages (Graveley *et al.* 2011).

Genomic and transcriptomic resources have been developed for a variety of species within the phylum Cnidaria (Moya *et al.* 2012; Barshis *et al.* 2013; Fuchs *et al.* 2014; Helm *et al.* 2013; Lehnert *et al.* 2012; Meyer *et al.* 2009, 2011; Polato *et al.* 2011; Shinzato *et al.* 2014; Soza‐Ried *et al.* 2010; Traylor-Knowles *et al.* 2011; Wenger and Galliot 2013; Sun *et al.* 2013; Meyer and Weis 2012; Lehnert *et al.* 2014), a diverse group of evolutionarily and ecologically significant species that range from hydroids (Class Hydrozoa) and jellyfish (Class Medusozoa) to sea anemones and corals (Class Anthozoa). Cnidarians are among early-diverging or basal metazoans and occupy a key position as a sister taxon to the bilaterians (Dunn *et al.* 2008). Many cnidarians play an important role in marine trophic cascades due to their mutualistic relationship with dinoflagellate species of the genus *Symbiodinium* that reside inside of cnidarian host cells. This relationship is based on nutritional exchange in which *Symbiodinium* spp. provide the cnidarian host with products from photosynthesis in return for inorganic nutrients and a stable, high-light environment (Davy *et al.* 2012). The paramount examples of this partnership are the reef-building corals, which form the trophic and structural foundation of productive and biodiverse coral reef ecosystems. Anthropogenic stressors, especially those associated with global climate change, are gravely threatening these reef ecosystems, including the corals themselves (Douglas 2003; Weis and Allemand 2009). Insight into the molecular mechanisms that underlie coral-dinoflagellate symbioses and their stress response to environmental perturbation is critical for future management and conservation of coral reef ecosystems.

To date, there are three publically available sequenced genomes from the Anthozoa: the symbiotic coral *Acropora digitifera* (Shinzato *et al.* 2011), symbiotic sea anemone *Aiptasia sp.* (Baumgarten *et al.* 2015) and the nonsymbiotic sea anemone, *Nematostella vectensis* (Putnam *et al.* 2007). These genomes have provided insight into the genomic complexity of cnidarians, furthering studies of gene evolution and function across basal metazoans (Poole and Weis 2014; Putnam *et al.* 2007; Shinzato *et al.* 2011; Marlow *et al.* 2009; Ryan *et al.* 2006; Hamada *et al.* 2013; Shinzato *et al.* 2012a, b; Wood-Charlson and Weis 2009; Dunn *et al.* 2008). Comparison of these genomes has revealed putative symbiosis-associated genes that may function in the onset and maintenance of cnidarian-dinoflagellate symbiosis (Meyer and Weis 2012). Annotated *de novo* transcriptomes, generated using next generation sequencing (NGS) [expressed sequence tags (ESTs), 454 pyrosequencing and Illumina HiSeq technologies], have been published for eight genera of anthozoans (Polato *et al.* 2011; Kenkel *et al.* 2013; Meyer *et al.* 2009; Traylor-Knowles *et al.* 2011; Lehnert *et al.* 2012; Pratlong *et al.* 2015; Shinzato *et al.* 2014; Vidal-Dupiol *et al.* 2013). These resources have been used in a variety of contexts, including the study of gene family evolution (Poole and Weis 2014), symbiosis-enhanced gene expression (Lehnert *et al.* 2014), and responses to environmental stressors such as elevated seawater temperature (Meyer *et al.* 2011; Kenkel *et al.* 2013), bacterial infection (Closek *et al.* 2014), and CO_2_-driven changes in seawater pH (Vidal-Dupiol *et al.* 2013). These studies are adding to earlier generation omics studies [EST studies, subtractive hybridization and cDNA microarrays (Meyer and Weis 2012)] and are providing information on the mechanisms of cnidarian-dinoflagellate symbiosis and coral bleaching, a stress response that results from the breakdown of the partnership (Weis 2008; Davy *et al.* 2012). Expression studies are therefore contributing not only to our basic understanding of cellular processes in cnidarians but also to our ability to link molecular responses with phenotypic change due to environmental perturbation.

The available anthozoan resources are limited in taxonomic diversity and dominated by a few genera from a narrow geographic range (Meyer and Weis 2012). In addition, many resources are from aposymbiotic (lacking dinoflagellate symbionts) samples or nonsymbiotic species, which limits the study of interplay between the two partners. One goal of this work is to increase the number and diversity of anthozoan resources for comparative, phylogenetic, and functional analyses.

In this study, we present transcriptomes from four anthozoans: the sea anemone *Anthopleura elegantissima* (Brandt 1835) and the corals *Fungia scutaria* (Lamarck 1801), *Montastraea cavernosa* (Linnaeus 1767), and *Seriatopora hystrix* (Dana 1846) in varying symbiotic states, life history stages, and geographic locations (Table 1). These species are of particular interest to investigations into the molecular mechanisms associated with the onset, maintenance, and breakdown of cnidarian-dinoflagellate symbioses. We highlight how these transcriptomes can be used in applications ranging from targeted gene searches to orthologous group predictions and phylogenomic analysis. In addition, we outline a method to screen for cross-contamination between sequencing libraries that can be broadly applied to other transcriptome studies.

**Table 1:** Collection sites, life history stages, and symbiotic states of the four anthozoans used for transcriptome assembly

Organism	Collection Site	Developmental Stage	Symbiotic State
*Anthopleura elegantissima*	Seal Rock, Oregon	Adult	Aposymbiotic
*Fungia scutaria*	Coconut Island, Hawaii	Larval	Aposymbiotic
*Montastraea cavernosa*	Florida Keys, Florida	Adult	Symbiotic
*Seriatopora hystrix*	Nanwan Bay, Taiwan	Adult	Symbiotic

## Materials and Methods

### Sample collection and RNA extraction

All four anthozoan species examined in this study engage in symbiosis with *Symbiodinium* spp., and therefore RNA extractions typically include contributions from the dinoflagellate symbionts at some level. Here, two samples (*M. cavernosa* and *S. hystrix*) were collected from symbiotic specimens and two samples (*F. scutaria* and *A. elegantissima*) were collected from nominally aposymbiotic stages or specimens (Table 1). Larvae of *F. scutaria* were reared in filtered seawater at the Hawaii Institute of Marine Biology following fertilization and development and remained symbiont-free during development (Schnitzler and Weis 2010). The aposymbiotic specimen of *A. elegantissima* was collected in that condition in the field.

Total RNA was extracted from *S. hystrix*, *F. scutaria*, and *A. elegantissma* using the following methods. *S. hystrix* tissue was stored in RNA*later* Stabilization Solution (Qiagen, CA) and RNA was extracted using the RNeasy Mini Kit (Qiagen, CA) according to the manufacturer’s protocol. Whole animal specimens of *A. elegantissima* (aposymbiotic) and *F. scutaria* (larvae) were collected, frozen in liquid nitrogen, and stored at −80°. RNA was extracted using a combination of the TRIzol RNA isolation protocol (Life Technologies, CA) and RNeasy Mini Kit (Qiagen, CA). The TRIzol protocol was used for initial steps up to and including the chloroform extraction. Following tissue homogenization, an additional centrifugation step was performed at 12,000 × *g* for 10 min to remove tissue debris. After the chloroform extraction, the aqueous layer was combined with an equal volume of 100% EtOH and the RNeasy Mini Kit was used to perform washes following the manufacturer’s protocol.

A core sample of *M. cavernosa* was collected, frozen in liquid nitrogen, and stored at −80°. Total RNA from *M. cavernosa* was extracted following a modified TRIzol protocol with a 12-M LiCl precipitation (Mazel *et al.* 2003). Briefly, the coral fragment was vortexed in TRIzol reagent for 15 min and then processed according to the manufacturer’s instructions through phase separation. To precipitate RNA, 0.25 ml of isopropanol and 0.25 ml of a high-salt solution (0.8 M sodium citrate and 1.2 M NaCl) per 1 ml of TRIzol used was added to the aqueous supernatant. The addition of the high-salt solution removes proteoglycan and polysaccharide contaminants. The solution was incubated at room temperature for 10 min and then centrifuged at 12,000 × *g* for 10 min at 4°. After centrifugation, the standard TRIzol protocol was followed through the ethanol wash. To remove PCR inhibitors of an unknown nature that are frequently encountered in coral samples, RNA was precipitated by adding an equal volume of 12 M LiCl and then was incubated for 30 min at −20°. The sample was centrifuged at 12,000 × *g* for 15 min at room temperature and washed with 75% EtOH (1 ml per 1 ml of TRIzol), followed by centrifugation at 7500 × *g* for 5 min at room temperature. The supernatant was removed and the RNA pellet was air-dried.

The extracted total RNA from each sample was DNase-treated using a TURBO DNA-Free Kit (Ambion, CA) to remove genomic DNA contamination. RNA quantity and quality were assessed using the NanoDrop ND-1000 UV-Vis Spectrophotometer (Thermo Scientific, MA) and gel electrophoresis.

### Preparation of sequencing libraries

Polyadenylated RNA was purified from 10 µg of total RNA using the Magnetic mRNA Isolation Kit (New England Biolabs, MA). First strand cDNA was synthesized using ProtoScript M-MuLV FS-cDNA Synthesis Kit (New England Biolabs, MA) according to the manufacturer’s protocol and modified oligonucleotides in Supporting Information, Table S1. Second strand synthesis was performed by incubating first-strand cDNA with 1× NEBNext Second Strand Synthesis Buffer (New England Biolabs, MA), 0.2 mM dNTPs, 15 units of *Escherichia coli* DNA ligase (New England Biolabs, MA), 75 units of *E. coli* DNA polymerase I (New England Biolabs, MA), and 3 units of RNase H (New England Biolabs, MA) for 2 hr at 16°. cDNA was purified using the GeneJet PCR Purification Kit (Fermentas, MA) and then fragmented using NEBNext dsDNA Fragmentase (New England Biolabs, MA) according to the manufacturer’s protocol, with the addition of 5 mM MgCl_2_ and 1 mg ml^−1^ BSA (New England Biolabs, MA). Fragmented cDNA was purified and the ends were repaired using NEB Quick Blunting Kit (New England Biolabs, MA) according to manufacturer’s protocol. The product was purified and A-tailed in a reaction with nuclease-free water, 1× NEB Standard *Taq* buffer (New England Biolabs, MA), 1 mM dATP, and 2 units of NEB Standard *Taq* (New England Biolabs, MA) at 68° for 2 hr. Tailed templates were ligated to double stranded adaptors prepared with oligonucleotides from the Illumina Customer Sequence Letter (version August 12, 2014, Illumina 2014; Table S1). Purified, tailed cDNA was combined with T4 DNA ligase buffer (New England Biolabs, MA), T4 DNA ligase (New England Biolabs, MA), and the double stranded adaptors, and the solution was incubated at 12° for at least 6 hr. Ligation products were purified and then amplified using custom sample-specific barcodes (“indices”) designed with a 3-bp minimum Hamming distance based on Illumina barcodes (Illumina 2014) (Table S1). PCR included template cDNA, Phusion Taq polymerase buffer (Thermo Scientific, MA), dNTPs, 5′ Illumina “i5” barcoding oligo, 3′ Illumina “i7” multiplex oligonucleotide, and Phusion High Fidelity Taq polymerase (Thermo Scientific, MA). Reactions were incubated at 98° for 30 sec, followed by 17–21 cycles of the following: 98° for 10 sec, 63° for 30 sec, and 72° for 1.5 min. Reactions were amplified for the minimum cycle number required to produce a visible product on a 1% agarose gel. PCR products were size-selected by excising the 350–550 bp fraction from a 2% agarose gel. Finally, size-selected sequencing libraries were extracted using the E.Z.N.A. Gel Extraction Kit (Omega Bio-Tek, GA).

### Sequencing, processing, and assembly

cDNA libraries were sequenced on Illumina HiSeq 2000 at University of Oregon’s Genomics Core Facility (Eugene, OR). All cDNA libraries were pooled on a single lane to produce 100-bp paired-end reads. Raw sequences were filtered using custom Perl scripts to remove uninformative (matching adaptors in Table S1, or poly-A tail) and low-quality reads (>20 positions with quality scores <20) (Meyer *et al.* 2011). All custom scripts used in this study are available online at GitHub (https://github.com/Eli-Meyer). The high-quality filtered reads were then assembled using default settings in Trinity v2.0.2, a de Bruijn graph-based assembler that uses paired-end data to reconstruct transcripts and group these into components intended to represent the collection of transcripts originating from a single gene (Grabherr *et al.* 2011).

### Functional annotation

To develop these assemblies as resources for functional studies, we assigned putative gene names and functional categories [Gene Ontology (GO) and Kyoto Encyclopedia of Genes and Genomes (KEGG)] to assembled transcripts based on sequence comparisons with online databases. All sequence comparisons were conducted using BLAST+ from National Center for Biotechnology Information (NCBI) (Package version 2.2.29) (Altschul *et al.* 1990). Gene names were assigned by comparing transcript sequences against UniProt protein sequence databases (SwissProt and TREMBL) using BLASTx with an expect value (E value) cutoff of 10^−4^. Each transcript was assigned a gene name based on its best match, excluding matches with uninformative names (*e.g.*, uncharacterized, unknown, or hypothetical). GO terms describing biological processes, molecular functions, and cellular components were assigned to each transcript based on GO-UniProt associations of its best match downloaded from the Gene Ontology website (Ashburner *et al.* 2000). KEGG orthology terms were assigned from single-directional best hit BLAST searches of each transcriptome on the KEGG Automatic Annotation Server (Moriya *et al.* 2007).

### Reference transcriptome databases

To enhance the utility of these resources for the coral research community, we have also developed searchable databases and made these publicly available on the author’s laboratory website hosted at Oregon State University (http://people.oregonstate.edu/∼meyere/index.html). Databases were produced using the open-source SQLite software library and can be queried directly using a publicly accessible web form. To demonstrate the utility of our searchable databases for rapidly identifying genes of interest, we searched each database for a few genes previously studied in cnidarians, including a cell adhesion molecule (sym32) (Reynolds *et al.* 2000), a cysteine biosynthesis enzyme [cystathionine β-synthase, (Cbs)] (Shinzato *et al.* 2011), and a fluorescent protein (GFP) (Mazel *et al.* 2003; Shinzato *et al.* 2012b; Smith-Keune and Dove 2008). For comparison with these simple text searches, we also conducted a more comprehensive search for each gene based on reciprocal BLAST. Representative sequences for each gene were obtained from the UniProt database (version 2014_09, downloaded October 20, 2014), and searched against each assembly using tBLASTn (bit-score ≥45). The matching transcripts were then reciprocally compared against UniProt using BLASTx. Reciprocal matches were evaluated at the level of gene names; transcripts identified by searching with a target gene (*e.g.*, B5T1L4, GFP from *Acropora millepora*) were accepted if they reciprocally matched a different gene with corresponding annotation (*e.g.*, Q9U6Y6, a GFP gene from *Anemonia manjano*).

### Evaluating gene content and completeness of assembly

An ideal reference transcriptome would include all genes present in the genome of an organism, but low or tissue-specific expression can lead to incomplete sampling of genes during cDNA library preparation. To evaluate the gene representation of our assemblies, we searched each assembly for sequence similarity with a core set of conserved eukaryotic genes (CEGMA) (Parra *et al.* 2007) and with gene models from sequenced anthozoan genomes: the coral *A. digitifera* (OIST: adi_v1.0.1) (Shinzato *et al.* 2011) and the anemone *N. vectensis* (assembly version: Nemve1) (Putnam *et al.* 2007). Sequence comparisons were conducted using NCBI’s BLASTx (Altschul *et al.* 1990) and bit-scores ≥50 were considered significant.

An ideal transcriptome assembly would also include complete transcripts as contiguous sequences or contigs, but variation in coverage and sequence characteristics lead to fragmented assemblies consisting of partial transcripts. To evaluate the effectiveness of our assemblies in reconstructing complete transcripts, we calculated the Ortholog Hit Ratio (OHR), a metric ranging from 0 to 1 that indicates the proportion of each gene included in the assembled transcript (O’Neil *et al.* 2010). Each assembly was compared to gene models from the *N. vectensis* genome using BLASTx to identify orthologs. We calculated OHR first with a relatively stringent approach (OHR_HITS_) as the proportion of each *N. vectensis* gene included within local alignments with assembled transcripts (high-scoring segment pairs in BLASTx output). Because this approach excludes divergent regions, we calculated OHR with an alternative and more inclusive approach (OHR_ORF_) as the ratio of the transcript’s longest ORF (in the BLASTx-defined reading frame) relative to the length of its corresponding *N. vectensis* protein. When multiple transcripts matched a single gene, we considered only the longest OHR. Distributions of maximum OHR scores and summary statistics were examined to evaluate the completeness of each assembly.

### Screening for biological contamination

**Figure 1:**
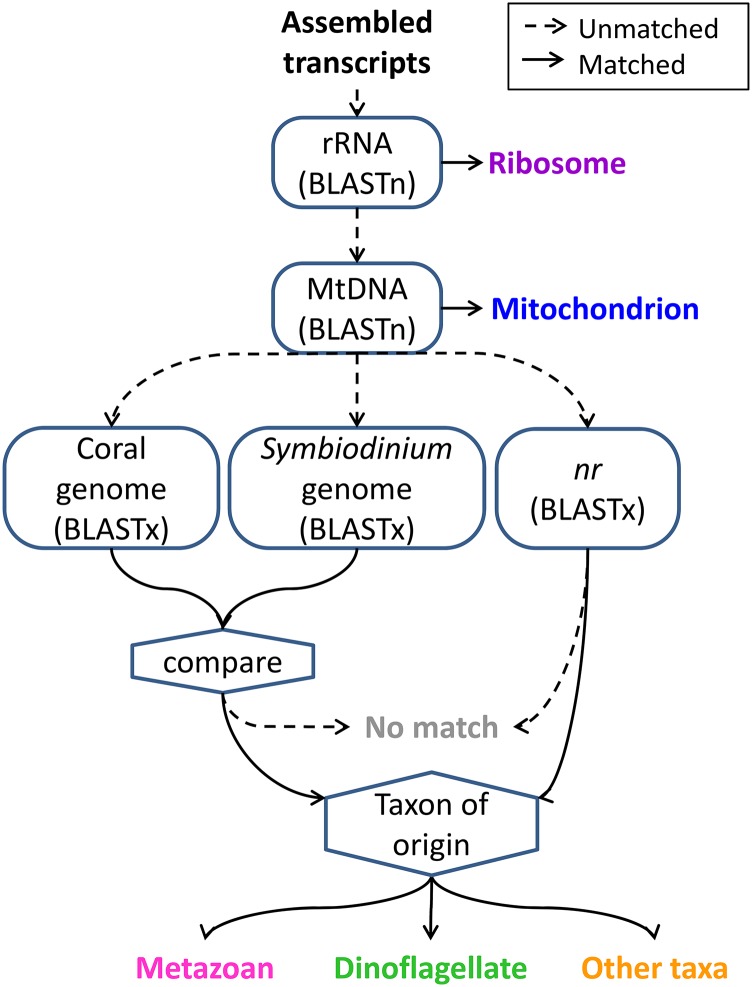
Annotation pipeline used to classify origins of each assembled transcript. A series of sequence comparisons was performed comparing each transcript against *N. vectensis* rRNA (SILVA: ABAV01023297, ABAV01023333), *A. tenuis* mitochondrial DNA (NCBI: NC_003522.1), *A. digitifera* and *S. minutum* gene models, and the NCBI nonredundant protein database (bit-score threshold of 45 for small databases; E value threshold of 10^−5^ for large databases). Transcripts were assigned to categories by evaluating their similarity to each database in the order shown (see *Materials and Methods* for details).

All species used in this study engage in symbiotic associations with intracellular dinoflagellate symbionts; therefore, RNA extracted from these specimens is expected to include contributions from both animal hosts and dinoflagellate symbionts. To evaluate these contributions we conducted a series of sequence comparisons aiming to identify the taxonomic origin of each transcript (Figure 1). Transcripts were compared with a series of sequence databases using BLAST v.2.2.29 with a bit-score threshold of 45. To identify transcripts derived from rRNA, each assembly was compared with cnidarian rRNA sequences using BLASTn. *N. vectensis* sequences were chosen for this purpose because they represent the most complete cnidarian sequences in the SILVA rRNA database (SILVA: ABAV01023297, ABAV01023333) (Quast *et al.* 2013). Transcripts were compared with a cnidarian mitochondrial genome using BLASTn; for this analysis, we chose the complete mitochondrial genome from *Acropora tenuis* (NCBI: NC_003522.1) (van Oppen *et al.* 2002). To identify the taxonomic origin of each transcript, sequences were compared with the NCBI nonredundant (nr) protein database (downloaded March 12, 2014) using BLASTx (E value ≤10^−5^) (Altschul *et al.* 1990). To avoid errors that might arise from the scarcity of cnidarian and dinoflagellate sequences in these databases, transcripts were compared with gene models from *Symbiodinium minutum* (clade B) (OIST: symbB.v1.2.augustus.prot) and *A. digitifera* (OIST: adi_v1.0.1_prot) using BLASTx. The taxonomic origin of each sequence was categorized as follows. First, transcripts matching rRNA or mitochondrial sequences were assigned to those categories. Transcripts matching *Symbiodinium* genes more closely than coral genes that did not return a metazoan hit as their best match in nr were assigned to the dinoflagellate category. Transcripts matching coral genes more closely than *Symbiodinium* genes that also matched metazoan records or lacked matches in nr were categorized as metazoan. Transcripts that showed conflicting results (metazoan in one database but nonmetazoan in the other) were categorized as “unknown.” Transcripts lacking any match to either coral or *Symbiodinium* genes were assigned based on taxonomic annotation of the best match in nr, if available. This series of decisions made it possible to classify each transcript based on origin (ribosomal, mitochondrial, other metazoan, dinoflagellate, or other taxa, which includes prokaryotes, uncertain, or no match).

### Screening for cross-contamination

During preliminary analysis of the transcriptome assemblies, we observed a few orthologs with unexpectedly high sequence similarity (>99%) among species.

Because cross-contamination could realistically occur at several different stages during multiplex library preparation and sequencing, we tested for evidence of cross-contamination in our transcriptome assemblies and developed a pipeline to eliminate contaminating sequences. To evaluate the extent of cross-contamination in our libraries, we mapped the cleaned reads used to produce each assembly against that assembly using the Trinity utility *align_and_estimate_abundance.pl* (Haas *et al.* 2013). We then compared all transcriptome libraries sequenced and prepared together using BLASTn to identify nearly identical sequences present in multiple assemblies (bit-score ≥100). This analysis identified many sequences occurring in multiple assemblies, which were highly abundant in one sample (consistent with this being their true origin) but were very low in abundance (<10-fold lower) in other assemblies (consistent with cross-contamination). To evaluate the level of sequence similarity expected among anthozoan transcriptomes, for comparison with the similarity observed among our assemblies, we compared publicly available transcript assemblies produced independently in different labs [*Pocillopora damicornis*, (Traylor-Knowles *et al.* 2011); *A. digitifera*, (Shinzato *et al.* 2011); and *A. millepora*, (Meyer *et al.* 2009)]. To eliminate putative cross-contaminants identified in our assemblies, we first compared assemblies using BLASTn to identify highly similar sequences (bit-score ≥100). We then estimated the abundance of each transcript in each assembly by mapping and counting reads from each library against the assembly produced from those reads using the Trinity utility *align_and_estimate_abundance.pl*. To identify and remove sequences that might result from cross-contamination, we categorized each transcript based on sequence similarity and relative expression in all other assemblies. Any transcripts with nearly identical matches in more than one assembly were assigned to the assembly in which each was most abundant if the sequence was at least 10-fold more abundant in that library than any others. Alternatively, transcripts found at comparable levels (<10-fold difference) in multiple assemblies were flagged as “unknown origin” and excluded from further analysis.

### Development of SSR markers

Simple sequence repeats (SSRs), also known as microsatellites, are sequences with repetitive 2–5 base pairs of DNA. These molecular markers have been widely used for studies of genome mapping, genetic linkage, and population structure. Although SSRs have largely been replaced with sequencing-based approaches for single nucleotide polymorphism (SNP) genotyping, in some situations they may still be the most practical option. To demonstrate the utility of transcriptome assemblies for SSR marker development and to identify SSR markers for the four species described here, we used a pipeline we have previously described for identifying SSRs in coral sequence data (Davies *et al.* 2013). In brief, sequences containing repetitive regions (≥30 bp, ≤15% deviation from perfect repeat structure, ≥30 bp flanking regions) were identified using RepeatMasker v3.2.9 (Smit *et al.* 1996) and then assembled using CAP3 to eliminate redundancy (Huang and Madan 1999). Target sequences were further screened for redundancy using BLASTn (Altschul *et al.* 1990) to identify unique targets within each repeat type (*e.g.*, AT, CCG, etc.). Finally, primer sequences flanking these SSRs were developed using Primer3 (Rozen and Skaletsky 1999), targeting regions 150–500 bp with 45–65% GC content.

### Identification of orthologous groups

To facilitate comparative studies of cnidarian gene sequences, and to demonstrate the utility of our transcriptome assemblies for phylogenetic analysis, we identified orthologous groups among the four transcriptomes generated in this study. We also compared these with sequence resources from other cnidarians and basal metazoans, including a marine sponge *Amphimedon queenslandica* (Srivastava *et al.* 2010), the hydrozoan *Hydra magnipapillata* (Chapman *et al.* 2010), the schyphozoan *Aurelia aurita* (Fuchs *et al.* 2014), and a variety of other anthozoans including *Aiptasia pallida* (Lehnert *et al.* 2012), *N. vectensis* (Putnam *et al.* 2007), *A. digitifera* (Shinzato *et al.* 2011), *Porites astreoides* (Kenkel *et al.* 2013), *P. damicornis* (Vidal-Dupiol *et al.* 2013), *Stylophora pistillata* (Karako-Lampert *et al.* 2014), *Orbicella faveolata* (formerly belonging to the genus *Montastraea*) (Budd and Stolarski 2011; DeSalvo *et al.* 2008), and *Pseudodiploria strigosa* (Table S2). These resources varied in the types of sequencing technologies used to create them, and this resulted in differing degrees of assembly completeness, ranging from whole genomes to EST libraries (Table S2). All resources were converted into candidate protein coding sequences using the package TransDecoder (transdecoder.sourceforge.net) that identifies open reading frames. Protein sequences were then processed with FastOrtho (enews.patricbrc.org/fastortho), an OrthoMCL-based program (Li *et al.* 2003) that performs an all-by-all BLAST of the input sequences (E value cutoff ≤10^−5^) and clusters orthologous groups with the Markov Cluster algorithm (Van Dongen 2000).

### Phylogenetic analysis

The four transcriptomes from this study and other sequence resources were used to infer phylogenetic relationships from commonly used markers and newly identified orthologs. The mitochondrial gene *cytochrome c oxidase 1* (COI) has been used to reconstruct the most comprehensive phylogeny of corals (Anthozoa, Scleractinia) (Kitahara *et al.* 2010), and mitochondrial sequences are commonly used to infer evolutionary relationships of the Cnidaria (Kitahara *et al.* 2010; Bridge *et al.* 1992; Kayal *et al.* 2013). Recent findings suggest, however, that a concatenated set of *NADH dehydrogenase* genes (ND 2, 4, and 5), called ND supergene, outperforms COI in metazoan datasets including in anthozoans (Havird and Santos 2014).

To investigate the effect of increased gene sampling on phylogenetic inferences, we compared phylogenetic trees constructed based on the widely used marker COI, the ND supergene, and the set of orthologs identified from a comparison of our transcriptomes with other cnidarian sequence resources. All taxa used in searches for orthologous groups were included and *A. queenslandica* served as the outgroup. The Transdecoder catalog of proteins for each organism was made into a local BLAST database. Then, the mitochondrial protein sequences of COI, ND2, ND4, and ND5 were found from BLASTx searches against our local databases, UniProt database, or NCBI database (Table S3 and Table S4). In some cases, mitochondrial genes were not recovered from the local protein databases, but they were found by tBLASTx to the original resources. These transcripts were instead translated using Expasy Translate Tool (http://web.expasy.org/translate/) under the “invertebrate mitochondrial” genetic code. Proteins sequences for COI, ND2, ND4, and ND5 were aligned using MAFFT v6.864b (Katoh *et al.* 2002). In some cases, the mitochondrial sequences were fragmented within a single database or recovered from two separate databases (Table S3 and Table S4). These fragments were aligned and manually combined to increase total alignment positions. Individual MAFFT alignments of ND2, ND4, and ND5 were concatenated into a single matrix in Mesquite (v. 3.02) (Maddison and Maddison 2015). Protein alignments of COI and the ND genes were run through ProtTest server (http://darwin.uvigo.es/software/prottest_server.html) (Abascal *et al.* 2005) to select the appropriate substitution rate model based on AIC and BIC criterion. Phylogenetic trees were constructed using maximum likelihood (ML) in RAxML v8.0.26 (Stamatakis 2014) under the MTZOA+G+F model (Rota-Stabelli *et al.* 2009). Optimal topology was selected based on ML scores from 500 replicate trees. Nodal support was assessed from 500 bootstrap replicates.

For phylogenomic reconstruction, the computational pipeline PhyloTreePruner (Kocot *et al.* 2013) was applied to orthologous groups with a minimum amino acid length of 100 from the 15 taxa identified in Table S2. PhyloTreePruner is a phylogenetic approach used to refine orthologous groups identified in programs like OrthoMCL by removing predicted paralogs resulting from gene duplication or splice variants through single gene-tree evaluation (Kocot *et al.* 2013). First, each group of orthologs was aligned using MAFFT v6.864b with 1000 iterations. Ambiguous or uninformative positions were removed from the alignment using Gblocks v0.91b (Castresana 2000). Then, single-gene ML trees for each group inferred with FastTree2 (Price *et al.* 2010) were screened for paralogy with PhyloTreePruner and the longest sequence for each taxon was retained. The pruned orthologous groups were then merged into a single matrix using FASconCAT v1.0 (Kück and Meusemann 2010). To examine the impact of missing data on tree topology, two trees were constructed. In the conservative tree, 14–15 taxa were sampled per ortholog for a total of 397 groups (73,833 unique alignment positions). The relaxed tree allowed more missing data, requiring only at least 10 taxa sampled per ortholog for a total of 2896 groups (535,413 unique alignment positions). For each dataset, ML trees were inferred with RAxML v8.0.26 using the WAG+GAMMA+F substitution model (Whelan and Goldman 2001). Topology for each tree was selected from 100 replicate trees, and nodal support values are based on 100 and 500 bootstrap replicates in the conservative and relaxed trees, respectively.

### Data availability

The data sets supporting the results of this article are available from the Sequence Read Archive at NCBI (Accession number: SRP063463), the Dryad Digital Repository (doi: 10.5061/dryad.3f08f), and the author’s website (http://people.oregonstate.edu/∼meyere/index.html).

## Results and Discussion

### Sequencing and *de novo* assembly

The four libraries described here were sequenced on Illumina HiSeq 2000 (each occupying 1/6th of a lane), yielding on average 26.3 million paired reads per library (range, 21.2–30.3; Table S5). A fraction of these (22% on average; range 14–28%) were removed during quality and adaptor filtering prior to assembly. Assembly of the remaining high-quality reads produced on average ∼170,000 transcripts. This is substantially higher than the number of genes in sequenced cnidarian genomes (23,677 in *A. digitifera*, 27,273 in *N. vectensis*), which likely results from redundancy, fragmentation in the assemblies, and biological contamination. Assemblies included many small contigs (on average, 47% were <400 bp) that were unlikely to provide significant matches, so for analyses based on sequence homology we considered only contigs ≥400 bp (average n = 91,792). For these core transcriptome datasets used for downstream analyses, the average length ranged from 1.1 to 1.7 kb and N_50_ ranged from 1.4 to 2.7 kb. These are slightly shorter than the expected size distribution for a complete cnidarian transcriptome (*e.g.*, average ∼1700 and N_50_ ∼2200 bp transcripts in the *A. digitifera* genome), suggesting incomplete assemblies. Assembly statistics of the four transcriptome references developed in this study are broadly comparable to previously published anthozoan transcriptomes (Moya *et al.* 2012; Shinzato *et al.* 2011, 2014; Abascal *et al.* 2005; Traylor-Knowles *et al.* 2011; Lehnert *et al.* 2012).

### Completeness of transcriptomes

**Figure 2:**
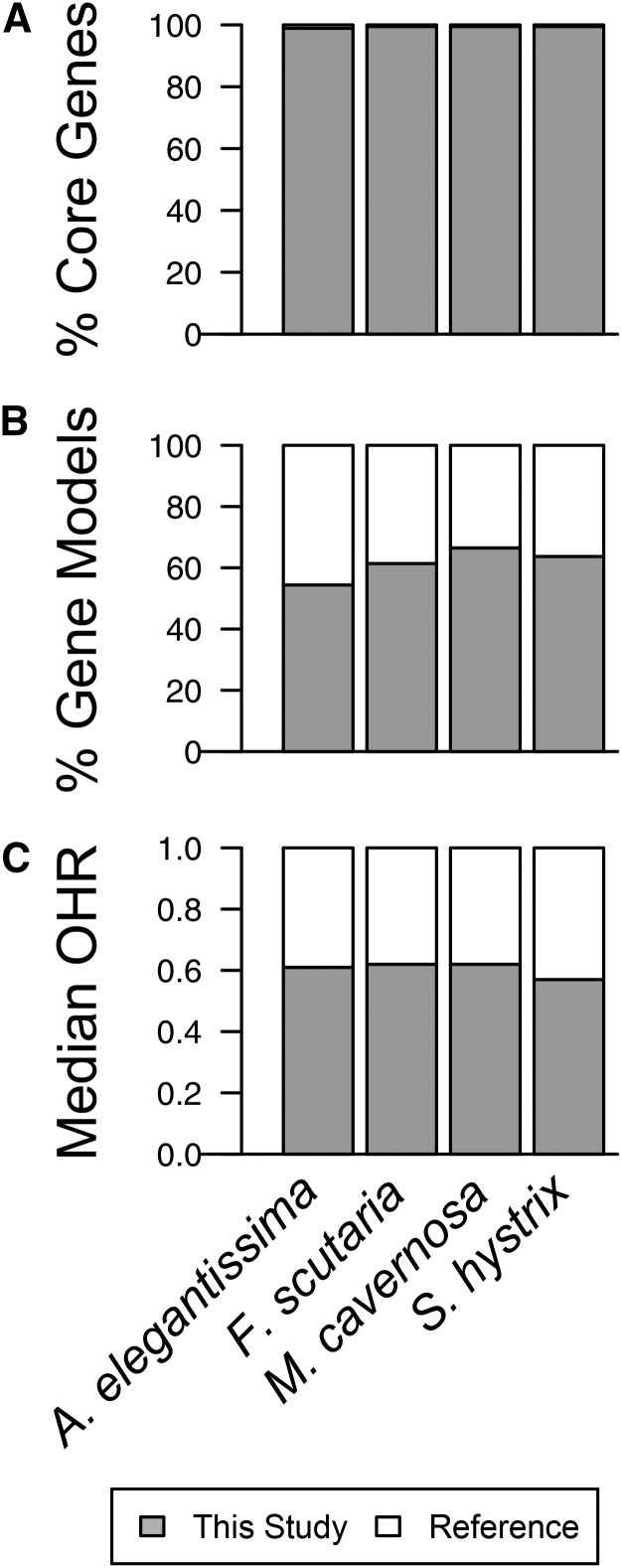
Three metrics used to evaluate gene representation and assembly of complete transcripts in *de novo* transcriptome assemblies. (A) Percent of core eukaryotic genes (CEGMA) identified in each assembly. (B) Percent of *A. digitifera* gene models with significant matches in each assembly. (C) Median proportion of each *N. vectensis* protein aligned with transcripts in each assembly (OHR_HITS_). Gray = our transcriptome assembly compared to the respective reference for each analysis.

To evaluate the completeness of the transcriptome assemblies from the perspective of gene content, we conducted sequence comparisons with conserved eukaryotic genes and gene models from sequenced relatives. The core eukaryotic genes (CEGMA) (Parra *et al.* 2007) are expected to be expressed in most eukaryotes (Nakasugi *et al.* 2013; Sanders *et al.* 2014) and are widely used to estimate transcriptome completeness. Sequence comparisons revealed matches for 453 (98.9%) of these conserved genes in *A. elegantissma* and 456 (99.5%) in *F. scutaria*, *M. cavernosa*, and *S. hystrix* (Figure 2A). For a more comprehensive view of gene representation, the transcriptomes were compared with gene models from sequenced relatives (the coral *A. digitifera* and the anemone *N. vectensis*). This analysis identified matches for more than 14,000 gene models in each genome (BLASTx, bit-score ≥50): 54–67% of gene models in *A. digitifera* (Figure 2B) and 48–49% in *N. vectensis*. This is comparable to the level of sequence similarity observed among anthozoans with completed genomes. BLASTp comparisons of predicted proteins from the *A. digitifera* and *N. vectensis* genomes using the same thresholds recover 35% and 42% of genes in the other genome. This is substantially lower than the optimistic estimates of representation based on CEGMA, perhaps reflecting essential functions and constitutive expression of these highly conserved genes. Comparisons with gene models of closely related taxa appear to provide a more conservative estimate of gene representation in transcriptome assemblies.

To evaluate the effectiveness of our assemblies in reconstructing complete transcripts, we calculated ortholog hit ratios (OHR) for each final assembly. This method estimates the amount of a *de novo* transcript contained in the best ortholog from a reference genome (O’Neil *et al.* 2010), ranging from 1 (for complete transcripts) to 0 (for transcript fragments). We calculated OHR based on sequence comparisons with *N. vectensis* gene models using two approaches. First, a relatively stringent analysis based on the proportion of each *N. vectensis* gene included in regions of local similarity (OHR_HITS_) produced median OHR of 63.8, 64.7, 65.7, and 58.0% for *A. elegantissma*, *F. scutaria*, *M. cavernosa*, and *S. hystrix*, respectively (Figure 2C). A more inclusive analysis based on the longest ORF (in BLAST defined frame) produced similar estimates (median OHR_ORF_: 67.4, 75.8, 77.2, and 60.3%, respectively). Each assembly included more than 5000 reasonably complete transcripts spanning at least 75% of the corresponding *N. vectensis* gene (range, 5262–6725). Overall, these comparisons with existing cnidarian sequence resources quantify the representation and completeness of our assemblies and provide a framework for comparison with other *de novo* assemblies. These estimates compare favorably with previous transcriptome completeness estimates for cnidarians (Sanders *et al.* 2014) and several invertebrates (O’Neil and Emrich 2013; Riesgo *et al.* 2012) using similar methods.

### Annotation of transcriptomes

Transcripts were annotated using BLAST homology searches against the UniProt databases. Approximately one-third of all transcripts matched records in UniProt (range, 30–40%) (Table S5). The relatively low fraction of sequences annotated is attributable in part to sequence lengths: on average, 21% of transcripts <400 bp in length were annotated as compared with 42% of transcripts 400–1000 bp in length and 78% of transcripts >1000 bp. Even among the longest transcripts (>1 kb), a substantial number of sequences lacked annotated matches in UniProt (range, 6647–12,090 sequences per assembly). This highlights the well-known bias in taxonomic composition of existing databases and the value of ongoing gene sequencing in underrepresented metazoan taxa for public sequence databases.

**Figure 3:**
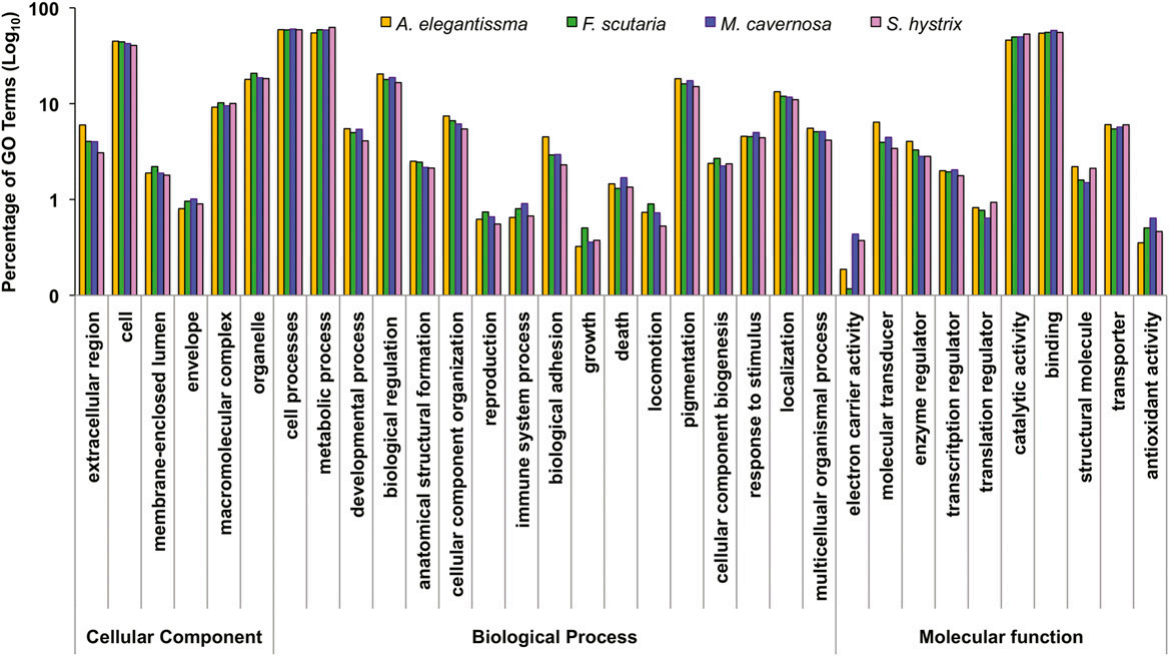
Distribution of functional categories (GO terms) in each transcriptome assembly. The percentage of transcripts with GO annotation for each category under the three main ontology domains was calculated for each assembly.

To categorize the biological functions inferred from sequence similarity, Gene Ontology (GO) terms were assigned to transcripts matching GO-annotated records in the UniProt database. This process identified functional annotation for 77% of transcripts with BLAST matches, providing tentative gene identities for a large number of sequences in each assembly (range, 32,299–47,547 transcripts; Table S5). Figure 3 shows the distribution of functional categories across the four transcriptomes, visualized using the Web Gene Ontology Annotation Plotting (WEGO) application. The GO terms were broadly distributed across the three domains and the percentages of sequences mapped to a given sub-ontology were highly similar for all species and comparable to other invertebrate transcriptomes (Riesgo *et al.* 2012; O’Neil *et al.* 2010; Lehnert *et al.* 2012; Moya *et al.* 2012; Polato *et al.* 2011; Shinzato *et al.* 2014; Stefanik *et al.* 2014; Traylor-Knowles *et al.* 2011). The similarities in functional distributions of assemblies prepared from diverse species, developmental stages, and symbiotic states highlight the constitutive expression of a broad set of genes in cnidarian transcriptomes. These core genes should facilitate comparative transcriptome studies by increasing the overlap among incomplete libraries.

**Figure 4:**
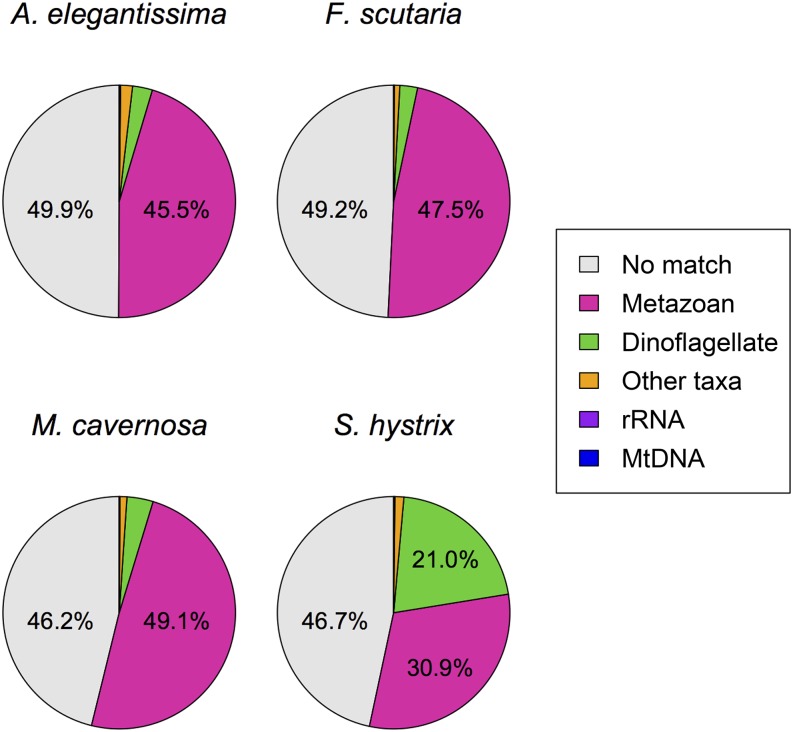
Predicted taxonomic origin of transcriptomes based on homology searches with BLAST. The percent of transcripts that were assigned to rRNA (purple), mtDNA (blue), dinoflagellate (green), metazoan (pink), other taxa (orange), and no match (gray) are shown.

To determine taxonomic origin for each transcript, we conducted a series of BLAST searches and filtering steps outlined in Figure 1. Because our assemblies were produced from symbiotic and aposymbiotic specimens, the transcriptomes contain genes not only from anthozoans but also from their associated microbial community. To investigate the relative contributions of these sources we classified each transcript based on sequence similarity (Figure 1). These analyses confirmed that metazoan sequences comprised the majority of each library as expected. Fortunately, only a small fraction of transcripts were derived from organelles (mitochondria and ribosomes): on average, 212 transcripts (range, 127–284) in each assembly matched rRNA (*N. vectensis*) and 30 transcripts (range, 16–54) matched the mitochondrial genome (*A. tenuis*). Notably, almost half of the transcripts in each assembly (range, 46.2–49.9%) lacked matches to coral or *Symbiodinium* spp. genes, or NCBI’s *nr* database (Figure 4), a range that is consistent with results from other anthozoan transcriptomes (Sun *et al.* 2013; Karako-Lampert *et al.* 2014; Polato *et al.* 2011; Traylor-Knowles *et al.* 2011). These "unknown" transcripts may represent lineage-specific genes ("taxonomically restricted genes") that require further characterization. Comparison with NCBI’s *nr* database revealed that the majority of sequences with matches in one or more databases (59–95%) matched a metazoan sequence better than any other taxon, suggesting they originated from the animal host rather than from dinoflagellate or prokaryotic symbionts. A negligible fraction of transcripts in each assembly (0.8–1.7%) was assigned to the “Other taxa” category, most of which matched either coral or *Symbiodinium* genes but were classified as “unknown” because of conflicting results in the *nr* search (*e.g.*, transcripts that matched *Symbiodinium* more closely than coral but whose best matches in *nr* were from metazoans).

The contribution of algal symbionts varied widely across samples. In nominally aposymbiotic samples of *F. scutaria* and *A. elegantissma*, 2.6% of transcripts on average were classified as dinoflagellate in origin (Figure 4), which may have resulted either from unexpected presence of symbionts at low abundance in these samples or from genes lacking orthologs in the *A. digitifera* reference. The symbiotic samples from *S. hystrix*, in contrast, showed a comparable abundance of transcripts classified as metazoan (61,369) and dinoflagellate in origin (41,724). Surprisingly, the *M. cavernosa* library that was similarly prepared from a symbiotic sample showed only 7278 transcripts from dinoflagellates (Figure 4). This striking contrast in *Symbiodinium* contributions from symbiotic specimens may have arisen from differing methods of RNA extraction. For *S. hystrix*, tissue was airbrushed off the coral skeleton directly into RNA*later* Stabilization Solution (Qiagen, CA), followed by complete tissue homogenization. In contrast, the *M. cavernosa* fragment was simply vortexed to disrupt tissue, without physical homogenization. Our findings suggest that omitting physical homogenization during lysis can minimize symbiont contamination for studies aiming to focus on the cnidarian host, whereas studies investigating both components may benefit from thorough homogenization during extraction. The gene names, functional categories, and putative origin of each transcript are annotated in Table S6, Table S7, Table S8, and Table S9.

### Gene searches of the database

The resulting annotations and sequences are available in a set of searchable databases hosted by Oregon State University (http://people.oregonstate.edu/∼meyere/index.html). To illustrate the utility of databases for cnidarian researchers targeting specific genes, we compared the effectiveness of simple text searches of the databases with reciprocal BLAST (RB) analysis, a more comprehensive approach that requires additional work by the end-user. Text searches targeting a few selected genes [cell adhesion molecule sym32, green fluorescent protein (GFP), and cystathionine β-synthase (Cbs)] produced comparable results as RB searches (Table S10). Text searches are obviously sensitive to query phrasing; the query “fluorescent” retrieves 51 putative GFP homologs, and functionally related synonyms (“GFP”, “chromoprotein”) retrieved an additional 10. Interestingly, the Cbs homologs identified in nominally aposymbiotic samples (*A. elegantissima* and *F. scutaria*) showed greater sequence similarity with *Symbiodinium* gene models than coral (*A. digitifera*) and were classified as dinoflagellate in our assignment procedure (Figure 1), whereas Cbs homologs in symbiotic samples (*M. cavernosa* and *S. hystrix*) included both metazoan and dinoflagellate transcripts. This unexpected observation of apparently dinoflagellate homologs of Cbs in nominally aposymbiotic samples is noteworthy because of their variable distribution among corals and possible roles in coral nutritional dependency on symbiosis (Shinzato *et al.* 2011). While this finding could be explained by undetected *Symbiodinium* harbored in these putatively aposymbiotic samples, the uncertainty introduced by these observations suggests that studies investigating the diversity of Cbs homologs across corals may require additional data (*e.g.*, *in situ* hybridization) to confirm transcript origins. Overall, the close agreement between rigorous computational searches and simple text searches in these examples illustrates the utility of our searchable online databases for rapidly identifying genes of interest in reference transcriptome assemblies.

### Novel SSR markers

Simple sequence repeats (SSRs or microsatellites) have been widely used to study genetic diversity, hybridization events, population structure, and connectivity in anthozoans (Concepcion *et al.* 2010; Fernandez-Silva *et al.* 2013; Selkoe and Toonen 2006; Ruiz-Ramos and Baums 2014), and can directly influence phenotypic traits by altering DNA replication, translation, and gene expression (Ruiz-Ramos and Baums 2014). SSR markers can be readily identified from *de novo* assemblies of NGS data and emerge as a side benefit in transcriptome assembly projects conducted for other purposes. We identified and developed primers for 52, 49, 73, and 75 candidate SSR markers in *A. elegantissma*, *F. scutaria*, *M. cavernosa*, and *S. hystrix*, respectively. Primer pairs for each species are listed in Table S11. For three of the species studied here, varying numbers of SSR markers are already available. Previous studies of *S. hystrix* have developed 10 SSR markers (Maier *et al.* 2001; Underwood *et al.* 2006) to study habitat partitioning within a single reef (Bongaerts *et al.* 2010), dispersal and recruitment patterns across multiple reefs (van Oppen *et al.* 2008; Kininmonth *et al.* 2010), and population changes associated with bleaching events (Underwood *et al.* 2007). Candidate SSR markers have been identified in *F. scutaria* (n = 118) from the coral host and dinoflagellate symbionts (Concepcion *et al.* 2010). SSR markers previously developed in *M. cavernosa* (Shearer *et al.* 2005; Serrano *et al.* 2014) have been used to investigate the population connectivity across depth and geographic distance (Serrano *et al.* 2014). The candidate SSR markers identified in this study provide additional markers for future studies along similar lines. To our knowledge, SSR markers have not been previously developed in *A. elegantissima*. Although the population structure of the host has not been described, analysis of their dinoflagellate symbionts revealed highly structured populations across their geographic range (Sanders and Palumbi 2011). The markers developed in this study for *A. elegantissima* provide tools to investigate population structure of the host across a similar range.

### Orthologous groups and phylogenomic reconstructions

With the increasing availability of transcriptomes and genomes, these datasets can now be mined to discover novel phylogenetic markers within Anthozoa and across the Cnidaria to resolve taxonomic uncertainties. Phylogenetic reconstruction of anthozoans has presented challenges because analyses based on morphology, life history, and molecular sequences have failed to adequately delineate taxonomic boundaries or evolutionary relationships (Daly *et al.* 2003). To date, molecular phylogenies for anthozoans have been based on one or a small number of markers including nuclear ribosomal 28S and 18S genes (Daly *et al.* 2003; Berntson *et al.* 1999), β*-tubulin* (Fukami *et al.* 2008), mitochondrial 16S (Daly *et al.* 2003), *cytochrome b* (Fukami *et al.* 2008), and COI (Kitahara *et al.* 2010; Fukami *et al.* 2008). Interestingly, mitochondrial sequences in anthozoans have extremely low mutation rates compared to the bilaterians and are therefore highly conserved, allowing for robust comparisons across distantly related taxa (van Oppen *et al.* 2002; Galtier *et al.* 2009). Therefore, the mitochondrial gene COI has been used recently to define evolutionary relationships among scleractinian corals (Kitahara *et al.* 2010; Fukami *et al.* 2008; Budd and Stolarski 2011) and to support the distinction of robust corals from the complex corals (Romano and Palumbi 1996).

One disadvantage to single gene phylogenetic inferences is that they suffer from weak phylogenetic signals, sensitivity to hidden paralogy, and spurious tree artifacts (Philippe *et al.* 2004). Despite these potential limitations, single gene trees have advanced the field of cnidarian systematics. However, polyphyly remains a problem among several anthozoan families when using both maximum likelihood and Bayesian analyses (Fukami *et al.* 2008; Budd and Stolarski 2011), which has led to recent shifts in taxonomic classification (Budd and Stolarski 2011). To expand beyond previous single-gene approaches, we performed phylogenomic analyses incorporating the four new transcriptomes and other available "omic" resources. By simultaneously increasing taxon and gene sampling, phylogenetic inference is expected to improve (Philippe *et al.* 2004) and may help resolve some of the challenges in reconstructing the evolutionary relationships of the Anthozoa and, more broadly, phylum Cnidaria.

For phylogenomic analysis, transcripts larger than 400 bp were converted to protein with TransDecoder and clustered into orthologous groups using FastOrtho. The number of assigned orthologous groups ranged from 14,144 to 21,147 for the four transcriptomes (Figure S1). Comparison of all four resulted in 6560 shared orthologs (Figure S1). The three coral species shared 2045 orthologs not found in anemones and the two most closely related corals (*M. cavernosa* and *F. scutaria*) shared 1682 orthologs absent from the other assemblies. By incorporating 11 additional taxa for phylogenomic analysis (Table S2), 443 orthologs were identified between all taxa. After setting a minimum protein length (100 amino acids), these orthologs were refined using the PhyloTreePruner analysis pipeline (Kocot *et al.* 2013). Filtering resulted in the identification of 397 orthologs for ≥14 taxa. These were used to construct a phylogenetic tree we termed “conservative” because loci with any missing data were excluded (Table S12).

Missing data are a commonly encountered problem in phylogenomic analyses, from either reduced transcript length or gene absence from a transcriptome (Philippe *et al.* 2004; Kocot *et al.* 2013; Roure *et al.* 2013). However, the sensitivity of phylogenetic inference to incomplete datasets is still under investigation, with mixed results from phylogenomic analyses on large but patchy supermatrices (Roure *et al.* 2013; Philippe *et al.* 2004). Because the resources in this study used for ortholog identification differed in completeness, ranging from EST libraries to complete genomes (Table S2), we tested the influence of missing data on our phylogenetic reconstruction. To investigate this, we lowered the required number of taxa per orthologous group to ≥10, which identified 2897 orthologs (Table S12). This second set was used to create the “relaxed” phylogeny, so called because loci with some missing data were included.

**Figure 5:**
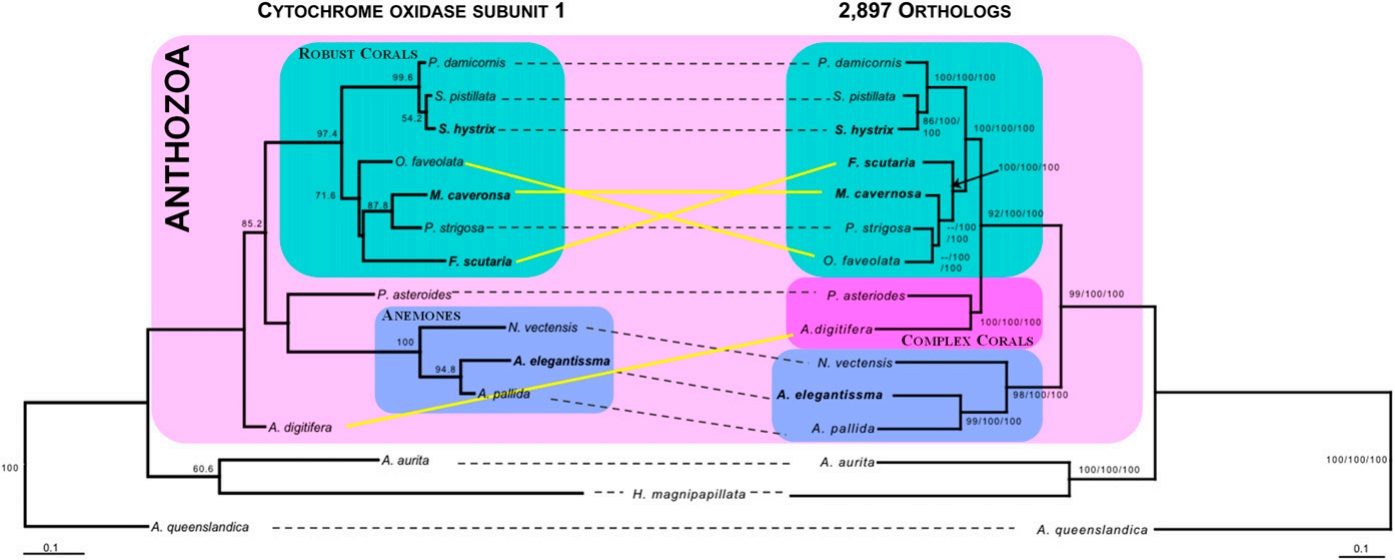
Discordance in maximum likelihood phylogenetic reconstruction of COI compared to a combined phylogeny of concatenated ND (2, 4, and 5) genes and two phylogenomic trees. The COI phylogeny is presented on the left and the combined phylogeny is presented on the right. Topology for the ND mitochondrial set, relaxed and conservative phylogenomic trees were nearly identical. Therefore, nodal support is summarized on the relaxed tree (right). Bootstrap support at the nodes from left to right represents ND gene set/relaxed/conservative. If topologies differed in the summary tree, then the nodal support is presented - - as next to the node. Yellow solid lines connect taxon with different positions and/or relationships between the two trees, whereas black dashed lines connect those with the same position and/or relationship. Reconstructions of groups in the class Anthozoa based on Kitahara *et al.* (2010) are highlighted in boxes: teal= robust corals; dark pink = complex corals; and light blue = anemones. The names of species used in this study are emphasized by bold font. Scale bars indicate the amino acid replacements per site.

Both maximum likelihood phylogenomic analyses reconstructed identical and strongly supported topologies (bootstrap = 100; Figure S2), demonstrating that our phylogenetic inference was insensitive to missing data (Figure 5). However, the relationship of the corals in the family Faviidae, containing *M. cavernosa*, *P. strigosa*, and *O. faveolata*, varied among the COI, ND supergene, and phylogenomic analyses. The mitochondrial ND supergene identified by Havird and Santos (2014) produced a phylogenetic tree nearly synonymous with the accepted cnidarian taxonomic relationships and phylogenomic analyses from this study (Kitahara *et al.* 2010), except for the placement of the *M. cavernosa* as sister taxon to *O. faveolata* and *P. strigosa*. The analysis of single gene COI resulted in a discordant phylogenetic topology (Figure 5), failing to reconstruct the complex coral clade (*P. astreoides* and *A. digitifera*), which was recovered by ND supergene, relaxed and conserved trees (Figure 5, Figure S2). In the COI tree, the placement of the *F. scutaria*, from the family Fungiidae, as sister taxon to *P. strigosa* and *M. cavernosa* from the family Faviidae, instead of *O. faveolata* is incongruent with current taxonomic placement (Figure 5) (Kitahara *et al.* 2010; Budd and Stolarski 2011). Furthermore, while the phylogenomic analyses placed *O. faveolata* as sister to *P. strigosa* with strong support (bootstrap = 100), this relationship was not recovered in either mitochondrial phylogeny (Figure S2). Overall, the tree topology from the phylogenomic analyses is consistent with accepted evolutionary relationships within Anthozoa (Budd and Stolarski 2011; Fukami *et al.* 2008; Kitahara *et al.* 2010).

### Conclusion

The annotated transcriptome assemblies developed in this study provide useful resources for genomic research in anthozoan species for which sequences resources were previously lacking. The searchable databases developed from these assemblies make it possible to rapidly identify genes of interest from each species. Our ortholog analysis demonstrates the feasibility of phylogenetic inference in corals using transcriptome assemblies from diverse stages and symbiotic states, highlighting a promising path toward resolving major uncertainties in the existing phylogeny of scleractinians. Future studies will benefit from the growing body of anthozoan sequence resources, including the four assemblies contributed in this study.

## 

## Supplementary Material

Supporting Information
