# Supplementary material for: De Novo Assembly and Characterization of Four Anthozoan (Phylum Cnidaria) Transcriptomes
Source: G3 (Bethesda). 2015 Sep 17;5(11):2441–52. doi: 10.1534/g3.115.020164 (PMC4632063; doi:10.1534/g3.115.020164)
Supplement: Supporting Information [file supp_5_11_2441__index.html]

De Novo Assembly and Characterization of Four Anthozoan (Phylum Cnidaria) Transcriptomes — Supporting Information 

# *De Novo* Assembly and Characterization of Four Anthozoan (Phylum Cnidaria) Transcriptomes

## Supporting Information for Kitchen *et al.*, 2015

**Files in this Data Supplement:**

- Supporting Information - Figures S1-S2 and Tables S1-S12 (PDF, 732 KB)
- Figure S1 - Venn diagram of shared orthologous groups. (PDF, 185 KB)
- Figure S2 - Individual maximum likelihood trees from COI, concatenated ND genes, relaxed and conservative taxon sampling across the whole transcriptomes and genomes. (PDF, 393 KB)
- Table S1 - Oligonucleotide primers used in sample preparation for Illumina sequencing. (PDF, 137 KB)
- Table S2 - Genomic and transcriptomic datasets used for ortholog identification and phylogenetic analyses. (PDF, 138 KB)
- Table S3 - Cytochrome oxidase subunit I (COI) sequences used in the phylogenetic analysis. (PDF, 149 KB)
- Table S4 - Supergene set of NADH dehydrogenase transcripts used in the phylogenetic analysis. (PDF, 138 KB)
- Table S5 - Transcriptome assembly and annotation statistics before and after a minimum transcript length was set to 400bp. (PDF, 198 KB)
- Table S10 - Comparison of gene searches by reciprocal BLAST or synonyms in online transcriptome databases. (PDF, 147 KB)
- Table S6 - Compiled annotation for *A. elegantissma* transcriptome including transcript ID, UniProt, GO and KEGG annotation, and ribosomal RNA, mitochondrial DNA or taxa origin from local and NCBI database searches. (.xls, 8 MB)
- Table S7 - Compiled annotation for *F. scutaria* transcriptome including transcript ID, UniProt, GO and KEGG annotation, and ribosomal RNA, mitochondrial DNA or taxa origin from local and NCBI database searches. (.xls, 11 MB)
- Table S8 - Compiled annotation for *M. cavernosa* transcriptome including transcript ID, UniProt, GO and KEGG annotation, and ribosomal RNA, mitochondrial DNA or taxa origin from local and NCBI database searches. (.xls, 10 MB)
- Table S9 - Compiled annotation for *S. hystrix* transcriptome including transcript ID, UniProt, GO and KEGG annotation, and ribosomal RNA, mitochondrial DNA or taxa origin from local and NCBI database searches. (.xls, 9 MB)
- Table S11 - Primers designed for potential SSR markers from *A. elegantissima*. (.xls, 71 KB)
- Table S12 - Orthologs used in relaxed (≥ 10 taxa) and conservative (≥ 14 taxa) phylogenomic analyses. (.xls, 1 MB)
