# Supplementary material for: De Novo Assembly and Characterization of Four Anthozoan (Phylum Cnidaria) Transcriptomes
Source: G3 (Bethesda). 2015 Sep 17;5(11):2441–52. doi: 10.1534/g3.115.020164 (PMC4632063; doi:10.1534/g3.115.020164)
Supplement: Supporting Information [file supp_g3.115.020164_020164SI.pdf]

G3: Genes | Genomes | Genetics

# *De novo* assembly and characterization of four anthozoan (phylum Cnidaria) transcriptomes

Sheila A. Kitchen [1] ^, Camerron M. Crowder ^, Angela Z. Poole, Virginia M. Weis and Eli Meyer

Department of Integrative Biology, Oregon State University, 3029 Cordley Hall, Corvallis, OR 97331, USA

^ Equal Contributors

[1]Corresponding Author: Department of Integrative Biology, 3029 Cordley Hall, Corvallis, OR 97330, USA. Email: kitchens@science.oregonstate.edu

**DOI: 10.1534/g3.115.020164**

**Table S1 Oligonucleotide primers used in sample preparation for Illumina sequencing.**

| Primer Name | Primer Source | Primer sequence (5' to 3') |
|---|---|---|
| PE Adapter | Illumina^ | ACACTCTTTCCCTACACGACGCTCTTCCGATCT |
| Multiplexing Index Read Primer | Illumina^ | GATCGGAAGAGCACACGTCTGAACTCCAGTCA |
| Adapter-i5 barcode | Illumina^ | AATGATACGGCGACCACCGAGATCTACAC[xxxxx] ACTCTTTCCCTACACGACGCTCTTCCGATCT |
| ILL-PCR Primer | Illumina^ | AATGATACGGCGACCACCGA |
| ILL-PCR Primer | Illumina^ | CAAGCAGAAGACGGCATACGA |
| Adapter-i7 barcode | Modified from Illumina^ | CAAGCAGAAGACGGCATACGAGAT[xxxxxx]GTGA CTGGAGTTCAGACGTGTGCTCTTCCGATCT |
| CA1-TS-YY | * | AGCAGTGGTATCAACGCAGAGTACYYGGG |
| CA1-20TVN | * | AAGCAGTGGTATCAACGCAGAGTACTTTTTTTTTT TTTTTTTTTTVN |
| CA1- | * | AAGCAGTGGTATCAACGCAGAGTAC |

^ Oligonucleotide sequences © 2007-2012 Illumina, Inc. All rights reserved, derivative works created by Illumina customers are authorized for use with Illumina instruments and products only. All other uses are strictly prohibited.

* Modified from TaKaRa Clontech Smart cDNA Synthesis Kit

**Table S2 Genomic and transcriptomic datasets used for ortholog identification and phylogenetic analyses.**

| Class, Order | Taxa | Dataset Type | Data Source | Publication |
|---|---|---|---|---|
| Demospongiae | *Amphimedon queenslandic* | Genome, WGS | Compagen | [58] |
| Anthozoa, Actinaria | *Aiptasia pallida* | Trascriptome, Illumina | Pringle Lab, Stanford | [13] |
| | *Anthopleura elegantissma* | Trascriptome, Illumina | This Study | |
| | *Nematostella vectensis* | Genome, WGS | Joint Genome Institute(JGI) | [26] |
| Anthozoa, Scleractinia- Complex | *Acropora digitifera* | Genome, 454 and Illumina | Okinawa Institute of Science and Technology (OIST) | [25] |
| | *Porites asteroides* | Transcriptome, 454 | Matz Lab, Univeristy of Texas, Austin | [34] |
| Anthozoa, Scleractinia- Robust* | *Pocillopora damicornis* | Transcriptome, Illumina | University of Perpignan Via Domitia | [35] |
| | *Seriatopora hystrix* | Transcriptome, Illumina | This Study | |
| | *Stylophora pistillata* | Transcriptome, 454 | Centre Scientifique de Monaco | [60] |
| | *Fungia scutaria* | Transcriptome, Illumina | This Study | |
| | *Montastraea caveronosa* | Transcriptome, Illumina | This Study | |
| | *Orbicella faveolata* | EST | Compagen | [62] |
| | *Pseudodiploria strigosa* | Transcriptome, Illumina | Meyer Lab, Oregon State University | |
| Hydrozoa | *Hydra magnipapillata* | Genome, WGS | Compagen | [59] |
| Scyphozoa | *Aurelia aurita* | Transcriptome, 454 | Compagen | [11] |

* Classification based on Kitahara et al. (2010) *PLoS ONE*
WGS= whole genome shotgun approach

**Table S3 Cytochrome oxidase subunit I (COI) sequences used in the phylogenetic analysis.**

| Taxon | Source Accession # | Source | Publication |
|---|---|---|---|
| *A. aurita* | AFV93084.1 | NCBI | Minxiao et al. (2012) |
| *A. digitifera* | cds.adi_v1.02255 | OIST | Shinzato et al. (2011) |
| *A. elegantissma* | comp60_c0_seq1 | This Study | |
| *A. pallida* | comp21186_c0_seq1 | Pringle Lab | Lehnert et al. (2012) |
| *A. queenslandic* | A2T558 (CL1690Contig1) | UniProt | Erpenbeck et al. (2007) |
| *F. scutaria* | comp17_c0_seq1 | This Study | |
| *H. vulgaris* | YP_002221538.1 | NCBI | Voigt et al. (2008) |
| *M. cavernosa* | comp5_c0_seq1 | This Study | |
| *N. vectensis* | ABF93433 | NCBI | Reitzel et al. (2008) |
| | jgi\|Nemve1\|239171\|estExt_fgenesh1_ | JGI | Putnam et al. (2007) |
| *O. faveolata* | AAS17032.1 | NCBI | Shearer and Coffroth (2008) |
| *P. asteroides* | AAS17049.1 | NCBI | Shearer and Coffroth (2008) |
| | contig01687 | Matz Lab | Kenkel et al. (2013) |
| *P. damicornis* | comp28171_c0_seq1 | University of Perpignan Via Domi | Vidal-Dupiol (2013) |
| *P. strigosa* | comp1018_c0_seq1 | This Study | |
| *S. hystrix* | comp6_c2_seq1 | This Study | |
| *S. pistillata* | SPI_contig00040 | Centre Scientifique de Monaco | Karako-Lampert et al. (2014 |

**Table S4 Supergene set of NADH dehydrogenase transcripts used in the phylogenetic analysis.**

| Taxon | Source Accession # | Source | Publication |
|---|---|---|---|
| **ND2** | | | |
| *A. aurita* | 03_aurelia_rc_finalASM_104 | Compagen | Fuchs et al. (2014) |
| | 03_aurelia_rc_finalASM_110 | Compagen | Fuchs et al. (2014) |
| *A. digitifera* | adi_v1.02258 | OIST | Shinzato et al. (2011) |
| *A. elegantissma* | comp6460_c0_seq1 | This Study | |
| *A. pallida* | comp50342_c0_seq1 | Pringle Lab | Lehnert et al. (2012) |
| *A. queenslandica* | CL1342Contig1 | Compagen | Srivastava et al. (2010) |
| *F. scutaria* | comp256_c0_seq1 | This Study | |
| *H. vulgaris* | B4F7M5 | UniProt | Voigt et al. (2008) |
| *M. cavernosa* | comp5_c0_seq1 | This Study | |
| *N. vectensis* | Q196M8 | UniProt | Medina et al. (2006) |
| *O. faveolata* | Q4G6D0 | UniProt | Fukami and Knowlton et al. (2005) |
| *P. asteroides* | contig06009 | Matz Lab | Kenkel et al. (2013) |
| | contig11255 | Compagen | Kenkel et al. (2013) |
| *P. damicornis* | comp28251_c0_seq1 | University of Perpignan Via Dom | Vidal-Dupiol (2013) |
| *P. strigosa* | comp270108_c0_seq1 | This Study | |
| *S. hystrix* | comp295_c0_seq1 | This Study | |
| *S. pistillata* | Spi_isotig05906 | Centre Scientifique de Monaco | Karako-Lampert et al. (2014) |
| | | | |
| **ND4** | | This Study | |
| *A. aurita* | 03_aurelia_rc_finalASM_35 | Compagen | Fuchs et al. (2014) |
| *A. digitifera* | adi_v1.02259 | OIST | Shinzato et al. (2011) |
| *A. elegantissma* | comp25_c0_seq1 | This Study | |
| *A. pallida* | comp21186_c0_seq1 | Pringle Lab | Lehnert et al. (2012) |
| *A. queenslandica* | CL131Contig1 | Compagen | Srivastava et al. (2010) |
| *F. scutaria* | comp189_c0_seq1 | This Study | |
| *H. vulgaris* | B4F7N2 | UniProt | Voigt et al. (2008) |
| *M. cavernosa* | comp5_c0_seq1 | This Study | |
| *N. vectensis* | Q196M6 | UniProt | Medina et al. (2006) |
| *O. faveolata* | Q4G6C7 | UniProt | Fukami and Knowlton (2005) |
| *P. asteroides* | contig10053 | Matz Lab | Kenkel et al. (2013) |
| | contig05864 | Matz Lab | Kenkel et al. (2013) |
| *P. damicornis* | comp51662_c0_seq3 | University of Perpignan Via Dom | Vidal-Dupiol (2013) |
| *P. strigosa* | comp41786_c0_seq1 | This Study | |
| | comp315167_c0_seq1 | This Study | |
| *S. hystrix* | comp66_c0_seq1 | This Study | |
| *S. pistillata* | Spi_isotig00545 | Centre Scientifique de Monaco | Karako-Lampert et al. (2014) |
| | | | |
| **ND5** | | | |
| *A. aurita* | Q06LF4 | UniProt | |
| *A. digitifera* | adi_v1.02255 | OIST | Shinzato et al. (2011) |
| | adi_v1.02256 | OIST | Shinzato et al. (2011) |
| *A. elegantissma* | comp6460_c0_seq1 | This Study | |
| *A. pulchella* | comp21186_c0_seq1 | Pringle Lab | Lehnert et al. (2012) |
| *A. queenslandica* | CL3360Contig1 | Compagen | Srivastava et al. (2010) |
| | CL883Contig1 | Compagen | Srivastava et al. (2010) |
| *F. scutaria* | comp7_c0_seq1 | This Study | |
| *H. vulgaris* | B4F7M6 | UniProt | Voigt et al. (2008) |
| *M. cavernosa* | comp5_c0_seq1 | This Study | |
| *N. vectensis* | jgi\|Nemve1\|76946\|gw.2815.3.1 | JGI | Putnam et al. (2007) |
| | jgi\|Nemve1\|71841\|gw.185.102. | JGI | Putnam et al. (2007) |
| *O. faveolata* | Q4G6D3 | UniProt | Fukami and Knowlton (2005) |
| *P. asteroides* | contig01415 | Matz Lab | Kenkel et al. (2013) |
| | contig08747 | Matz Lab | Kenkel et al. (2013) |
| *P. damicornis* | comp61190_c2_seq3 | University of Perpignan Via Dom | Vidal-Dupiol (2013) |
| | comp69518_c0_seq1 | University of Perpignan Via Dom | Vidal-Dupiol (2013) |
| *P. strigosa* | comp1791_c0_seq1 | This Study | |
| | comp251320_c0_seq1 | This Study | |
| *S. hystrix* | comp295_c1_seq1 | This Study | |
| | comp6_c2_seq1 | This Study | |
| *S. pistillata* | Spi_isotig04735 | Centre Scientifique de Monaco | Karako-Lampert et al. (2014) |
| | Spi_contig00075 | Centre Scientifique de Monaco | Karako-Lampert et al. (2014) |

**Table S5 Transcriptome assembly and annotation statistics before and after a minimum transcript length was set to 400bp.**

| | *A. elegantissma* | *F. scutaria* | *M. cavernosa* | *S. hystrix* |
|---|---|---|---|---|
| Total number of raw sequencing reads | 30,316,700 | 21,206,956 | 26,333,520 | 27,499,904 |
| Total number reads after quality filtering | 23,811,719 | 18,215,908 | 20,457,388 | 19,900,626 |
| Total number of contigs | 142,934 | 155,914 | 200,223 | 198,572 |
| Average contig length | 855 | 903 | 1,038 | 671 |
| Maximum contig length | 58,996 | 37,748 | 73,708 | 56,757 |
| Minimum contig length | 201 | 201 | 201 | 201 |
| n50 of all contigs | 1,505 | 1,619 | 2,192 | 969 |
| Total number of longest components | 69,930 | 65,978 | 88,472 | 136,303 |
| Total number of longest subcomponents | 71,676 | 67,679 | 89,931 | 138,577 |
| Number of transcripts with UniProt annotati | 57,227 | 60,715 | 77,581 | 60,456 |
| Number of transcripts with GO annotation | 43,911 | 46,203 | 59,926 | 46,404 |
| Number of transcripts with KEGG annotatio | 28,743 | 8,892 | 37,454 | 12,635 |
| | | | | |
| Total number of contigs > 400bp | 75,594 | 86,489 | 109,987 | 95,097 |
| Average contig length > 400bp | 1,367 | 1,402 | 1,663 | 1,098 |
| n50 > 400bp | 1,963 | 2,038 | 2,699 | 1,363 |
| Number of transcripts with UniProt annotati | 42,567 | 45,797 | 61,616 | 42,374 |
| Number of transcripts with GO annotation | 32,657 | 34,882 | 47,547 | 32,299 |

## Tables S6-S9

Available for download as Excel files at www.g3journal.org/lookup/suppl/doi:10.1534/g3.115.020164/-/DC1

**Table S6.** Compiled annotation for *A. elegantissma* transcriptome including transcript ID, UniProt, GO and KEGG annotation, and ribosomal RNA, mitochondrial DNA or taxa origin from local and NCBI database searches.

**Table S7.** Compiled annotation for *F. scutaria* transcriptome including transcript ID, UniProt, GO and KEGG annotation, and ribosomal RNA, mitochondrial DNA or taxa origin from local and NCBI database searches.

**Table S8.** Compiled annotation for *M. cavernosa* transcriptome including transcript ID, UniProt, GO and KEGG annotation, and ribosomal RNA, mitochondrial DNA or taxa origin from local and NCBI database searches.

**Table S9.** Compiled annotation for *S. hystrix* transcriptome including transcript ID, UniProt, GO and KEGG annotation, and ribosomal RNA, mitochondrial DNA or taxa origin from local and NCBI database searches.

**Table S10 Comparison of gene searches by reciprocal BLAST or synonyms in online transcriptome databases.** Bit-score cutoffs were set to 45 and taxonomic annotations were designated based on our taxonomic screen (Figure 1).

| Gene | Search Term or UniProt Accession # | *A. elegantissma* | *F. scutaria* | *M. cavernosa* | *S. hystrix* |
|---|---|---|---|---|---|
| Sym32 | Sym32 | 1 | 0 | 0 | 0 |
| | Q9NH96 | 1 | 0 | 0 | 0 |
| Cystathionine β Synthase (CBS) | Cystathionine beta- | 7 | 6 | 2 | 18 |
| | T2MGI5 | 6 | 6 | 1 | 17 |
| Green Fluorescent Protein | fluor | 3 | 8 | 28 | 12 |
| | GFP | 0 | 0 | 0 | 9 |
| | chromoprotein | 1 | 0 | 0 | 0 |
| | B5T1L4 | 3 | 4 | 16 | 7 |

## Tables S11-S12

Available for download as Excel files at www.g3journal.org/lookup/suppl/doi:10.1534/g3.115.020164/-/DC1

**Table S11.** Primers designed for potential SSR markers from *A. elegantissima*

**Table S12.** Orthologs used in relaxed (≥ 10 taxa) and conservative (≥ 14 taxa) phylogenomic analyses.

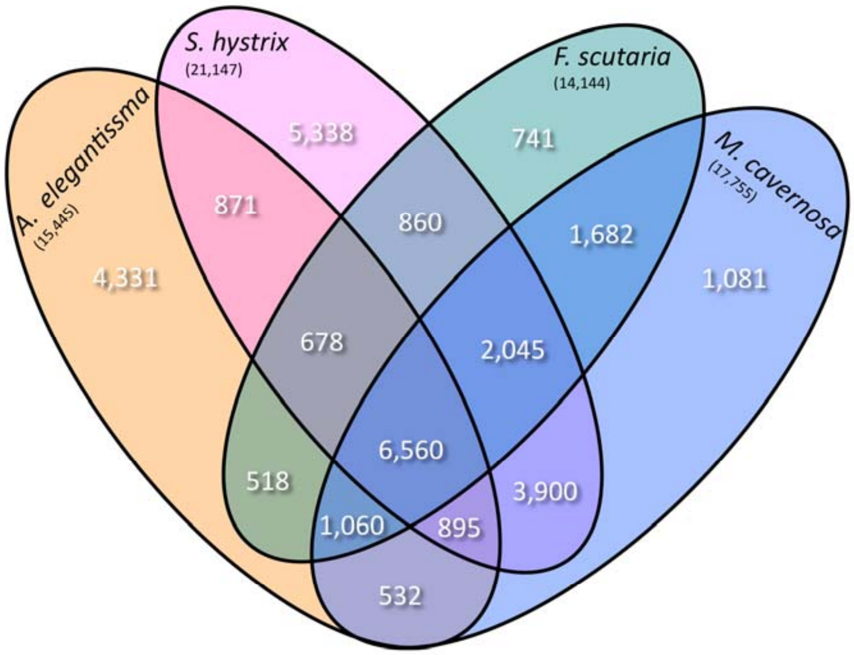


**Figure S1 Venn diagram of shared orthologous groups.** Comparision of the orthologous groups identified with FastOrtho from the four transcriptomes in this study. Total orthologous groups for each transcriptome are in parenthetical notation under the species name. *S. hystrix* and *M. cavernosa* shared the most orthologs (3,900) followed by *F. scutaria* and *M. cavernosa* (1,682).

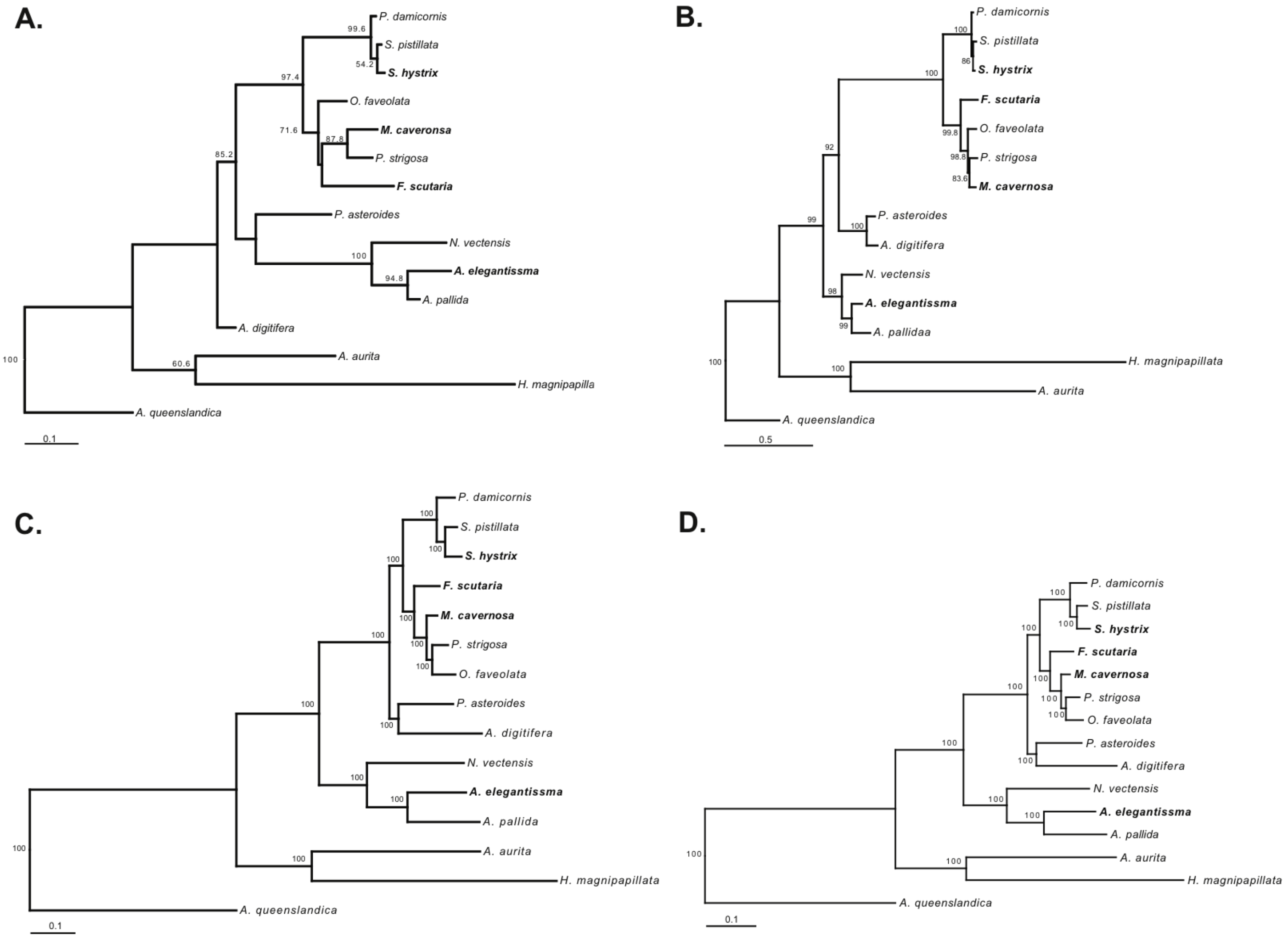


**Figure S2 Individual maximum likelihood trees from COI, concatenated ND genes, relaxed and conservative taxon sampling across the whole transcriptomes and genomes.** The optimal COI (A), ND genes (B), relaxed (C) and conservative (D) phylogenies are presented with nodal support from 500 bootstrap replicates, except for the relaxed with 100 bootstrap replicates. The four transcriptomes from this study are highlighted by bold font. The scale bar beneath each tree indicates the amino acid substitutions per site.
