# Supplementary material for: De Novo Assembly and Characterization of Four Anthozoan (Phylum Cnidaria) Transcriptomes
Source: G3 (Bethesda). 2015 Sep 17;5(11):2441–52. doi: 10.1534/g3.115.020164 (PMC4632063; doi:10.1534/g3.115.020164)
Supplement: Supporting Information [file supp_g3.115.020164_FigureS1.pdf]

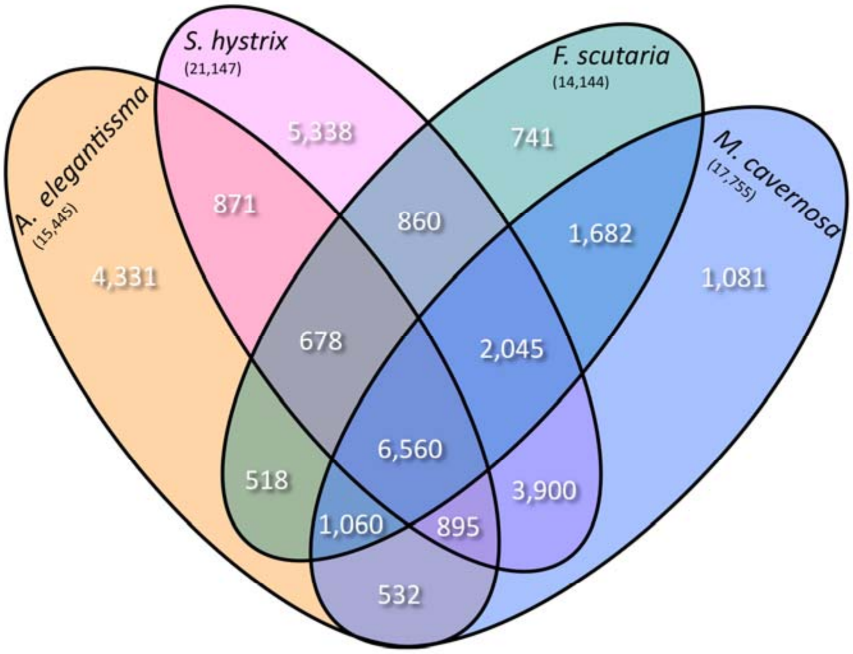

Figure S1 Venn diagram of shared orthologous groups. Comparision of the orthologous groups identified with FastOrtho from the four transcriptomes in this study. Total orthologous groups for each transcriptome are in parenthetical notation under the species name. *S. hystrix* and *M. cavernosa* shared the most orthologs (3,900) followed by *F. scutaria* and *M. cavernosa* (1,682).
