# Supplementary material for: De Novo Assembly and Characterization of Four Anthozoan (Phylum Cnidaria) Transcriptomes
Source: G3 (Bethesda). 2015 Sep 17;5(11):2441–52. doi: 10.1534/g3.115.020164 (PMC4632063; doi:10.1534/g3.115.020164)
Supplement: Supporting Information [file supp_g3.115.020164_FigureS2.pdf]

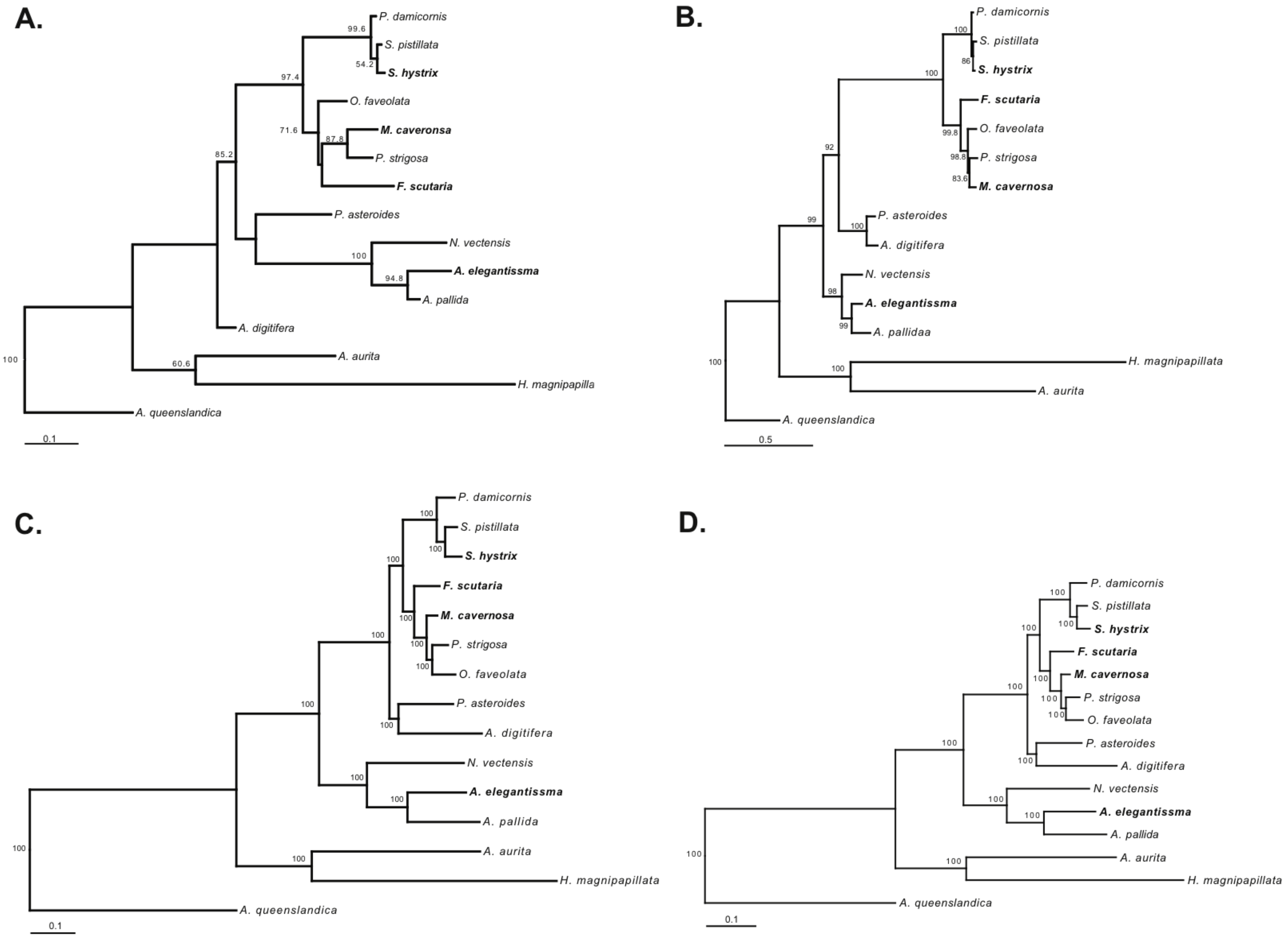

Figure S2 Individual maximum likelihood trees from COI, concatenated ND genes, relaxed and conservative taxon sampling across the whole transcriptomes and genomes. The optimal COI (A), ND genes (B), relaxed (C) and conservative (D) phylogenies are presented with nodal support from 500 bootstrap replicates, except for the relaxed with 100 bootstrap replicates. The four transcriptomes from this study are highlighted by bold font. The scale bar beneath each tree indicates the amino acid substitutions per site.
