# Supplementary material for: De Novo Assembly and Characterization of Four Anthozoan (Phylum Cnidaria) Transcriptomes
Source: G3 (Bethesda). 2015 Sep 17;5(11):2441–52. doi: 10.1534/g3.115.020164 (PMC4632063; doi:10.1534/g3.115.020164)
Supplement: Supporting Information [file supp_g3.115.020164_TableS1.pdf]

Table S1 Oligonucleotide primers used in sample preparation for Illumina sequencing.

Primer Name	Primer Source	Primer sequence (5' to 3')
PE Adapter	Illumina^	ACACTCTTTCCCTACACGACGCTCTTCCGATCT
Multiplexing Index Read Primer	Illumina^	GATCGGAAGAGCACACGTCTGAACTCCAGTCA
Adapter-i5 barcode	Illumina^	AATGATACGGCGACCACCGAGATCTACAC[xxxxx] ACTCTTTCCCTACACGACGCTCTTCCGATCT
ILL-PCR Primer	Illumina^	AATGATACGGCGACCACCGA
ILL-PCR Primer	Illumina^	CAAGCAGAAGACGGCATACGA
Adapter-i7 barcode	Modified from Illumina^	CAAGCAGAAGACGGCATACGAGAT[xxxxxx]GTGA CTGGAGTTCAGACGTGTGCTCTTCCGATCT
CA1-TS-YY	*	AGCAGTGGTATCAACGCAGAGTACYYGGG
CA1-20TVN	*	AAGCAGTGGTATCAACGCAGAGTACTTTTTTTTTT TTTTTTTTTTVN
CA1-	*	AAGCAGTGGTATCAACGCAGAGTAC

^ Oligonucleotide sequences © 2007-2012 Illumina, Inc. All rights reserved, derivative works created by Illumina customers are authorized for use with Illumina instruments and products only. All other uses are strictly prohibited.

* Modified from TaKaRa Clontech Smart cDNA Synthesis Kit
