# Supplementary material for: De Novo Assembly and Characterization of Four Anthozoan (Phylum Cnidaria) Transcriptomes
Source: G3 (Bethesda). 2015 Sep 17;5(11):2441–52. doi: 10.1534/g3.115.020164 (PMC4632063; doi:10.1534/g3.115.020164)
Supplement: Supporting Information [file supp_g3.115.020164_TableS2.pdf]

Table S2 Genomic and transcriptomic datasets used for ortholog identification and phylogenetic analyses.

Class, Order	Taxa	Dataset Type	Data Source	Publication
Demospongiae	*Amphimedon queenslandic*	Genome, WGS	Compagen	[58]
Anthozoa, Actinaria	*Aiptasia pallida*	Trascriptome, Illumina	Pringle Lab, Stanford	[13]
	Anthopleura elegantissma	Trascriptome, Illumina	This Study	
	Nematostella vectensis	Genome, WGS	Joint Genome Institute(JGI)	[26]
Anthozoa, Scleractinia- Complex	*Acropora digitifera*	Genome, 454 and Illumina	Okinawa Institute of Science and Technology (OIST)	[25]
	Porites asteroides	Transcriptome, 454	Matz Lab, Univeristy of Texas, Austin	[34]
Anthozoa, Scleractinia- Robust*	*Pocillopora damicornis*	Transcriptome, Illumina	University of Perpignan Via Domitia	[35]
	Seriatopora hystrix	Transcriptome, Illumina	This Study	
	Stylophora pistillata	Transcriptome, 454	Centre Scientifique de Monaco	[60]
	Fungia scutaria	Transcriptome, Illumina	This Study	
	Montastraea caveronosa	Transcriptome, Illumina	This Study	
	Orbicella faveolata	EST	Compagen	[62]
	Pseudodiploria strigosa	Transcriptome, Illumina	Meyer Lab, Oregon State University	
Hydrozoa	*Hydra magnipapillata*	Genome, WGS	Compagen	[59]
Scyphozoa	*Aurelia aurita*	Transcriptome, 454	Compagen	[11]

* Classification based on Kitahara et al. (2010) *PLoS ONE*

WGS= whole genome shotgun approach
