# Supplementary material for: De Novo Assembly and Characterization of Four Anthozoan (Phylum Cnidaria) Transcriptomes
Source: G3 (Bethesda). 2015 Sep 17;5(11):2441–52. doi: 10.1534/g3.115.020164 (PMC4632063; doi:10.1534/g3.115.020164)
Supplement: Supporting Information [file supp_g3.115.020164_TableS3.pdf]

Table S3 Cytochrome oxidase subunit I (COI) sequences used in the phylogenetic analysis.

Taxon	Source Accession #	Source	Publication
A. aurita	AFV93084.1	NCBI	Minxiao et al. (2012)
A. digitifera	cds.adi_v1.02255	OIST	Shinzato et al. (2011)
A. elegantissma	comp60_c0_seq1	This Study	
A. pallida	comp21186_c0_seq1	Pringle Lab	Lehnert et al. (2012)
A. queenslandic	A2T558 (CL1690Contig1)	UniProt	Erpenbeck et al. (2007)
F. scutaria	comp17_c0_seq1	This Study	
H. vulgaris	YP_002221538.1	NCBI	Voigt et al. (2008)
M. cavernosa	comp5_c0_seq1	This Study	
N. vectensis	ABF93433	NCBI	Reitzel et al. (2008)
	jgi|Nemve1|239171|estExt_fgenesh1_	JGI	Putnam et al. (2007)
O. faveolata	AAS17032.1	NCBI	Shearer and Coffroth (2008)
P. asteroides	AAS17049.1	NCBI	Shearer and Coffroth (2008)
	contig01687	Matz Lab	Kenkel et al. (2013)
P. damicornis	comp28171_c0_seq1	University of Perpignan Via Domi	Vidal-Dupiol (2013)
P. strigosa	comp1018_c0_seq1	This Study	
S. hystrix	comp6_c2_seq1	This Study	
S. pistillata	SPI_contig00040	Centre Scientifique de Monaco	Karako-Lampert et al. (2014
