# Supplementary material for: De Novo Assembly and Characterization of Four Anthozoan (Phylum Cnidaria) Transcriptomes
Source: G3 (Bethesda). 2015 Sep 17;5(11):2441–52. doi: 10.1534/g3.115.020164 (PMC4632063; doi:10.1534/g3.115.020164)
Supplement: Supporting Information [file supp_g3.115.020164_TableS4.pdf]

Table S4 Supergene set of NADH dehydrogenase transcripts used in the phylogenetic analysis.

Taxon	Source Accession #	Source	Publication
**ND2**			
A. aurita	03_aurelia_rc_finalASM_104	Compagen	Fuchs et al. (2014)
	03_aurelia_rc_finalASM_110	Compagen	Fuchs et al. (2014)
A. digitifera	adi_v1.02258	OIST	Shinzato et al. (2011)
A. elegantissma	comp6460_c0_seq1	This Study	
A. pallida	comp50342_c0_seq1	Pringle Lab	Lehnert et al. (2012)
A. queenslandica	CL1342Contig1	Compagen	Srivastava et al. (2010)
F. scutaria	comp256_c0_seq1	This Study	
H. vulgaris	B4F7M5	UniProt	Voigt et al. (2008)
M. cavernosa	comp5_c0_seq1	This Study	
N. vectensis	Q196M8	UniProt	Medina et al. (2006)
O. faveolata	Q4G6D0	UniProt	Fukami and Knowlton et al. (2005)
P. asteroides	contig06009	Matz Lab	Kenkel et al. (2013)
	contig11255	Compagen	Kenkel et al. (2013)
P. damicornis	comp28251_c0_seq1	University of Perpignan Via Dom	Vidal-Dupiol (2013)
P. strigosa	comp270108_c0_seq1	This Study	
S. hystrix	comp295_c0_seq1	This Study	
S. pistillata	Spi_isotig05906	Centre Scientifique de Monaco	Karako-Lampert et al. (2014)
**ND4**		This Study	
A. aurita	03_aurelia_rc_finalASM_35	Compagen	Fuchs et al. (2014)
A. digitifera	adi_v1.02259	OIST	Shinzato et al. (2011)
A. elegantissma	comp25_c0_seq1	This Study	
A. pallida	comp21186_c0_seq1	Pringle Lab	Lehnert et al. (2012)
A. queenslandica	CL131Contig1	Compagen	Srivastava et al. (2010)
F. scutaria	comp189_c0_seq1	This Study	
H. vulgaris	B4F7N2	UniProt	Voigt et al. (2008)
M. cavernosa	comp5_c0_seq1	This Study	
N. vectensis	Q196M6	UniProt	Medina et al. (2006)
O. faveolata	Q4G6C7	UniProt	Fukami and Knowlton (2005)
P. asteroides	contig10053	Matz Lab	Kenkel et al. (2013)
	contig05864	Matz Lab	Kenkel et al. (2013)
P. damicornis	comp51662_c0_seq3	University of Perpignan Via Dom	Vidal-Dupiol (2013)
P. strigosa	comp41786_c0_seq1	This Study	
	comp315167_c0_seq1	This Study	
S. hystrix	comp66_c0_seq1	This Study	
S. pistillata	Spi_isotig00545	Centre Scientifique de Monaco	Karako-Lampert et al. (2014)
**ND5**			
A. aurita	Q06LF4	UniProt	
A. digitifera	adi_v1.02255	OIST	Shinzato et al. (2011)
	adi_v1.02256	OIST	Shinzato et al. (2011)
A. elegantissma	comp6460_c0_seq1	This Study	
A. pulchella	comp21186_c0_seq1	Pringle Lab	Lehnert et al. (2012)
A. queenslandica	CL3360Contig1	Compagen	Srivastava et al. (2010)
	CL883Contig1	Compagen	Srivastava et al. (2010)
F. scutaria	comp7_c0_seq1	This Study	
H. vulgaris	B4F7M6	UniProt	Voigt et al. (2008)
M. cavernosa	comp5_c0_seq1	This Study	
N. vectensis	jgi|Nemve1|76946|gw.2815.3.1	JGI	Putnam et al. (2007)
	jgi|Nemve1|71841|gw.185.102.	JGI	Putnam et al. (2007)
O. faveolata	Q4G6D3	UniProt	Fukami and Knowlton (2005)
P. asteroides	contig01415	Matz Lab	Kenkel et al. (2013)
	contig08747	Matz Lab	Kenkel et al. (2013)
P. damicornis	comp61190_c2_seq3	University of Perpignan Via Dom	Vidal-Dupiol (2013)
	comp69518_c0_seq1	University of Perpignan Via Dom	Vidal-Dupiol (2013)
P. strigosa	comp1791_c0_seq1	This Study	
	comp251320_c0_seq1	This Study	
S. hystrix	comp295_c1_seq1	This Study	
	comp6_c2_seq1	This Study	
S. pistillata	Spi_isotig04735	Centre Scientifique de Monaco	Karako-Lampert et al. (2014)
	Spi_contig00075	Centre Scientifique de Monaco	Karako-Lampert et al. (2014)
