# Supplementary material for: De Novo Assembly and Characterization of Four Anthozoan (Phylum Cnidaria) Transcriptomes
Source: G3 (Bethesda). 2015 Sep 17;5(11):2441–52. doi: 10.1534/g3.115.020164 (PMC4632063; doi:10.1534/g3.115.020164)
Supplement: Supporting Information [file supp_g3.115.020164_TableS5.pdf]

Table S5 Transcriptome assembly and annotation statistics before and after a minimum transcript length was set to 400bp.

	A. elegantissma	*F. scutaria*	*M. cavernosa*	*S. hystrix*
Total number of raw sequencing reads	30,316,700	21,206,956	26,333,520	27,499,904
Total number reads after quality filtering	23,811,719	18,215,908	20,457,388	19,900,626
Total number of contigs	142,934	155,914	200,223	198,572
Average contig length	855	903	1,038	671
Maximum contig length	58,996	37,748	73,708	56,757
Minimum contig length	201	201	201	201
n50 of all contigs	1,505	1,619	2,192	969
Total number of longest components	69,930	65,978	88,472	136,303
Total number of longest subcomponents	71,676	67,679	89,931	138,577
Number of transcripts with UniProt annotati	57,227	60,715	77,581	60,456
Number of transcripts with GO annotation	43,911	46,203	59,926	46,404
Number of transcripts with KEGG annotatio	28,743	8,892	37,454	12,635
Total number of contigs > 400bp	75,594	86,489	109,987	95,097
Average contig length > 400bp	1,367	1,402	1,663	1,098
n50 > 400bp	1,963	2,038	2,699	1,363
Number of transcripts with UniProt annotati	42,567	45,797	61,616	42,374
Number of transcripts with GO annotation	32,657	34,882	47,547	32,299
