# Supplementary material for: De Novo Assembly and Characterization of Four Anthozoan (Phylum Cnidaria) Transcriptomes
Source: G3 (Bethesda). 2015 Sep 17;5(11):2441–52. doi: 10.1534/g3.115.020164 (PMC4632063; doi:10.1534/g3.115.020164)
Supplement: Supporting Information [file supp_g3.115.020164_TableS10.pdf]

Table S10 Comparison of gene searches by reciprocal BLAST or synonyms in online transcriptome databases. Bit-score cutoffs were set to 45 and taxonomic annotations were designated based on our taxonomic screen (Figure 1).

**Gene**	**Search Term or UniProt Accession #**	***A. elegantissma***	***F. scutaria***	***M. cavernosa***	***S. hystrix***
Sym32	Sym32	1	0	0	0
	Q9NH96	1	0	0	0
Cystathionine β Synthase (CBS)	Cystathionine beta-	7	6	2	18
	T2MGI5	6	6	1	17
Green Fluorescent Protein	fluor	3	8	28	12
	GFP	0	0	0	9
	chromoprotein	1	0	0	0
	B5T1L4	3	4	16	7
